# Differences in cardiovascular risk factors associated with sex and gender identity, but not gender expression, in young, healthy cisgender adults

**DOI:** 10.3389/fcvm.2024.1374765

**Published:** 2024-09-05

**Authors:** Jennifer S. Williams, Elise Wiley, Jem L. Cheng, Jenna C. Stone, William Bostad, Joshua M. Cherubini, Martin J. Gibala, Ada Tang, Maureen J. MacDonald

**Affiliations:** ^1^ Vascular Dynamics Lab, Department of Kinesiology, McMaster University, Hamilton, ON, Canada; ^2^ School of Rehabilitation Science, McMaster University, Hamilton, ON, Canada; ^3^ Human Performance Lab, Department of Kinesiology, McMaster University, Hamilton, ON, Canada

**Keywords:** sex differences, sex and gender-based analysis, flow-mediated dilation, pulse wave velocity, cardiovascular disease risk, arterial stiffness, endothelial function, blood pressure

## Abstract

**Background:**

Sex differences exist in cardiovascular disease risk factors including elevated blood pressure and arterial stiffness, and decreased endothelial function in males compared to females. Feminine gender expression may be associated with elevated risk of acute coronary syndrome. However, no study has investigated the associations between sex, gender identity, and gender expression and cardiovascular disease risk factors in young adults.

**Methods:**

One hundred and thirty participants (22 ± 3 years) underwent assessments of hemodynamics, arterial stiffness [pulse wave velocity (PWV)], and brachial artery endothelial function (flow-mediated dilation; %FMD). Participants completed a questionnaire capturing sex category (50 male/80 female), gender identity category (49 men/79 women/2 non-binary), and aspects of gender expression assessed by the Bem Sex Role Inventory-30 (39 androgynous/33 feminine/29 masculine/29 undifferentiated). Sex/gender identity category groups were compared using unpaired *t*-tests and gender expression groups compared using one-way ANOVAs.

**Results:**

Resting systolic and mean arterial pressure (*p* < 0.01) were elevated in males vs. females. Central PWV was elevated in males [median (interquartile range): 6.4 (1.8) vs. 5.8 (2.2) m/s, *p* = 0.02]; however, leg and arm PWV were not different between sexes. %FMD was elevated in males vs. females, after accounting for a larger baseline artery diameter in males (8.8 ± 3.3% vs. 7.2 ± 3.1%, *p* = 0.02); since the majority of participants were cisgender, the same results were found examining gender identity (men vs. women). There were no differences across gender expression groups (*p* > 0.05).

**Conclusions:**

Sex/gender identity category, but not gender expression, influence cardiovascular risk factors (blood pressure, arterial stiffness, endothelial function) in cisgender adults; further research is needed in gender-diverse populations.

## Introduction

Cardiovascular disease (CVD) is the leading cause of death worldwide (1–3). There are known sex differences in its pathophysiology and associated traditional and novel risk factors (4–6). For example, CVD progression and mortality are accelerated in males compared to females, and the first myocardial infarction occurs on average approximately 9 years earlier in males (7). Approximately 80% of this age-related difference is attributed to a deleterious cardiovascular risk profile in males earlier in life owing to factors such as smoking, hypertension, and diabetes (7). However, women tend to have a higher burden of disease and disability after the fifth decade of life; this burden is partly associated with the menopause transition (4, 6) and loss of the cardioprotective effects of the sex hormone estrogen (8, 9). This burden may also be attributed to social effects of aging, namely, increased stress associated with role strain with work and home (e.g., caregiving) responsibilities and stressful life events manifesting in mid-life (10).

Early risk factors for cardiovascular disease can predict future CVD risk (11) and follow similar age- and sex-related patterns of decline (9, 12, 13). Two important variables are endothelial function, measured by brachial artery flow-mediated dilation (FMD), and arterial stiffness, measured by pulse wave velocity (PWV). For example, a recent large multi-site trial identifying age and sex differences in brachial artery FMD found that while females have a higher relative FMD early in life compared to males, females experience a more rapid decline in FMD across the aging lifespan (12). This may be attributed to changes in hormone levels across the menopause transition, namely, the influence of 17β-estradiol (12). This study also found that males have a larger brachial artery diameter, regardless of age, which has been suggested to mask the capacity for the artery to dilate, thereby exacerbating the rapid decline observed in females (12). In contrast, research from our lab in young, healthy participants (age: 22 ± 3 years) identified sex differences in brachial artery FMD, such that FMD was elevated in males as compared to females when the influence of larger artery diameter in males was accounted for using allometric scaling (14). Accounting for sex differences in resting brachial artery diameter may contribute to the discrepant study results. However, other factors, such as differences in resting blood pressure (BP) or cardiorespiratory fitness, may also influence purposed sex differences in FMD (15, 16).

Arterial stiffness appears to be higher in males compared to females early in life, until middle age when arterial stiffness equalizes across sex. The effect is potentially associated with the menopause transition and the role of 17β-estradiol (17) and/or differences in anatomical growth patterns (17, 18), where stiffness rapidly increases in females (19). Our laboratory has found that local stiffness measured as carotid artery compliance was elevated in young males compared to females (20). While this study did not observe sex differences in central and peripheral arterial stiffness, measured via PWV (20), other studies have observed elevated central and peripheral arterial stiffness levels in males compared to females (21, 22). However, prior research examining the impact of sex on arterial stiffness and endothelial function did not consider other factors that may be underlying these sex differences. These include cardiorespiratory fitness, resting hemodynamics such as systolic blood pressure (SBP), and other intersections of identity, such as gender identity and expression.

We recently found that less than 1% of cardiovascular exercise physiology research has considered gender as a factor or variable that may differentially impact responses and that published studies only examined gender identity and not other constructs such as gender expression or roles (23). In contrast to sex categories, which mainly examines sex assigned at birth (i.e., male, female, intersex) and sex-related factors such as anatomy, chromosomes, and sex hormones, gender considers the socially constructed identity, expression, roles, and institutional structures and policies (24, 25) that act independently and/or synergistically to influence health (26, 27). Gender identity categories are commonly used in biomedical and clinical research, characterizing men, women, and gender-diverse individuals, including non-binary individuals (25, 26). Gender expression refers to how an individual portrays gendered personality traits, expressed on fluid continuums of masculine and feminine gender traits (28). Importantly, an individual's gender expression may not be in alignment with the stereotypically normative expression of their gender identity; for example, an individual identifying as a woman may not express high levels of feminine gender expression. As such, gender identity category and expression are examined as independent gender-related variables.

Gender expression, along with gender roles, may contribute to stress-related impacts on the cardiovascular system, such as the stress associated with caregiving, psychosocial stressors, and role strain/balance between work and home life (29–32); however, there are few studies that have examined gender identity category and expression on cardiovascular risk factors. For example, one study found that anger expression and control were associated with impaired cardiovascular health indices (i.e., blood pressure, blood lipids) in men, but not in women (33). Another study found that feminine gender roles and expression may play a role in the recurrence of acute coronary syndrome in young individuals, suggesting that examining gender expression and roles alongside sex and gender identity is of clinical importance (34). Further emphasizing the lack of research on gender and cardiovascular outcomes, a recent review by our group focused on the intersections of aging and sex/gender influences on cardiovascular indices also noted the lack of research examining gender (35). Similarly, a recent narrative review by Seeland et al. and the VascAgeNet Gender Expert Group points to the importance of examining both sex- and gender-related factors in vascular research and aging (36). We are not aware of any study to date that has examined the influence of gender identity and expression on early risk factors for CVD, including endothelial function or arterial stiffness, in young adults.

As the first study to investigate the associations between sex and gender and early risk indicators for cardiovascular disease in a young adult population, and aligned with recent calls for sex- and gender-based analysis in research (4, 24, 25, 37, 38), the following objectives and hypotheses were developed for this study:

1. Primary objective: To determine the association of sex category, gender identity category, and gender expression with endothelial function (measured using brachial artery FMD) and arterial stiffness (measured using PWV) in young, healthy adults.
  a. We hypothesized that males and men would have elevated FMD when baseline arterial diameter is considered, and this sex and gender difference may be attributed to higher cardiorespiratory fitness levels in males and men, compared to females and women, respectively.
  b. Similarly, we hypothesized that PWV would be higher in males and men compared to females and women, respectively (39).
  c. We also hypothesized that there would be a blunted cardiovascular profile (i.e., lower FMD and higher PWV) in individuals classified as feminine, aligned with prior clinical research on acute coronary syndrome in middle-aged adults (34).
2. Secondary objective: To investigate the relationships between feminine and masculine gender expression scores, cardiorespiratory fitness, central PWV, and FMD.
  a. We hypothesized that cardiorespiratory fitness would be associated with increased FMD and decreased central PWV (15, 16).
  b. We also hypothesized that higher feminine scores would be associated with decreased FMD and elevated central PWV.

## Materials and methods

### Participant recruitment and ethics

One hundred and thirty male and female participants between the ages of 18 and 45 years were recruited from the McMaster University and Hamilton communities through posters, online advertisements, and word of mouth. Participants were recruited from July 2021 to February 2023. A sample size calculation estimated that 50 participants (25 males, 25 females) would be needed to find sex differences in scaled %FMD, based on previous work in our lab (14) (%FMD: male: 9.0 ± 2.6%, female: 6.5 ± 2.1%, *α* ≤ 0.05, power: 95%; independent *t*-test in G*Power). However, given that no study had previously examined gender expression in a young, healthy cohort to base power calculations on, and given that a 1% change in %FMD is clinically relevant (11) and the robust change in clinical risk observed previously (34), we considered a 2% scaled FMD difference between gender expression groups (masculine, feminine, androgynous, undifferentiated; Δ2% change between feminine and masculine groups) to be meaningful. We determined that 120 participants were required to detect a significant difference assuming an SD of 2%, an alpha level of 0.05, and a power of 90% based on a one-way analysis of variance (ANOVA) in G*Power. This study was approved by the Hamilton Integrated Research Ethics Board (#14884) and conforms to the standards set by the *Declaration of Helsinki*, apart from registration of this study in a database. Participants completed a medical screening form to determine eligibility and provided written informed consent before participating in the study.

Participants were included if they were between the ages of 18 and 45 years, self-reported as healthy, non-smoking, and were located near Hamilton, Ontario, Canada. Participants were excluded if they reported cardiovascular or metabolic diseases, were pregnant or within the last year, were taking vasoactive medication (e.g., beta-blockers, ACE inhibitors, diuretics), or were smokers. Participants who had previously participated in a study in our laboratory within the last year and agreed to be contacted for future studies were recruited (*n* = 149), of which 130 agreed to participate. Potential participants who had been recruited using existing trials were re-consented to have their data included for this specific research question. Most participants (*n* = 114) were recruited prospectively through five existing studies that had embedded the demographic questionnaire into their study design and used lab-wide standardized methods for the relevant outcomes. A small number of participants (*n* = 16) were recruited retrospectively after completion of a study and asked to complete the questionnaire through a virtual medium (Zoom, San Jose, CA, USA).

### Study protocol

All study visits took place in the Vascular Dynamics Laboratory at McMaster University in a temperature- (23.3°C) and humidity-controlled (15%), quiet room. Prior to commencing the study, participants attended a familiarization visit to become familiar with the lab environment, assess eligibility, and complete consent forms and medical screening forms. Following recruitment, anthropometric information, including age, height, and weight to assess body mass index (kg/m^2^), was collected during the familiarization visit. Participants were also familiarized to the FMD test as described below.

Following the familiarization visit, participants took part in a vascular assessment session and a cardiorespiratory fitness exercise test. The cardiorespiratory fitness test was completed in close temporal proximity to the vascular assessment session, either in the week prior to the vascular assessment visit during the familiarization session (*n* = 64), immediately after on the same day (*n* = 46), or within 3 days following the vascular assessment visit (*n* = 20). Prior to the vascular assessment session, participants completed at least a 6 h overnight fast, 12 h without alcohol or caffeine, 24 h without moderate to vigorous physical activity, and 12 h without the use of prescription or non-prescription medications (i.e., anti-inflammatory and pain medications). Participants were tested during the morning hours to control for diurnal variation in endothelial function (40).

#### Demographic questionnaire

Participants were asked to complete a demographic questionnaire asking for information about their sex category (i.e., sex assigned at birth), gender identity category and gender expression, ethnicity, and race. Ethnicity and race were collected using questionnaires developed by the Governments of Canada (Canadian Census) (41) and Ontario (Ontario data standards for collection of race) (42), respectively. A two-step sex and gender question asked about sex at birth (options: female, male, intersex, prefer not to answer) and then about gender identity (woman, man, gender diverse/gender fluid, two-spirit, non-binary, prefer to self-describe, prefer not to answer), as a widely used question in clinical research settings (43, 44). Cisgender individuals were defined as those participants for whom their sex category corresponded with their gender identity category (i.e., female sex at birth, woman gender identity), while transgender individuals were defined as those participants for whom their sex category was different than their gender identity (i.e., female sex at birth, man gender identity). While these terms have been used to operationalize sex and gender identity categories in this study, we recognize that sex/gender contexts are evolving, and new language and research tools may better describe sex/gender categories in future. Finally, gender expression was assessed using the Bem Sex Role Inventory 30-item questionnaire (BSRI-30) that has been used previously in a university population, with high scores of internal consistency (*α* = 0.78–0.86) and moderate-to-high scores of test–re-test reliability across short (e.g., 4 weeks: *r* = 0.76–0.94) or long-term periods (e.g., 4 years: 0.56–0.+68) (28, 45–48). This version of the BSRI asks participants to identify with 30 traits from the BSRI according to a 7-point Likert-type scale from “1—Never or almost never true” to “7—Almost Always true” (Supplementary Table S1). Each trait was previously categorized as “masculine,” “feminine,” or “neutral,” with 10 traits in each category. A mean score for masculine (BSRI-masculine) and feminine (BSRI-feminine) was calculated based on the average of the 10 traits in each category. The median BSRI-masculine (4.40) and BSRI-feminine (5.20) scores were determined from the overall study population (*n* = 130), and each participant was categorized according to the following criteria:

Feminine: High BSRI-Feminine (≥5.20), Low BSRI-Masculine (<4.40);
Masculine: High BSRI-Masculine (≥4.40), Low BSRI-Feminine (<5.20);
Androgynous: High BSRI-Masculine (≥4.40) and BSRI-Feminine (≥5.20); and
Undifferentiated: Low BSRI-Masculine (<4.40) and BSRI-Feminine (<5.20).

Use of internal population-defined medians was chosen over the use of a 4.0 split or using Bem's original reported medians to reflect the gender expression unique to this population, such as generational or geographical differences in gender expression, supported by previous work (49, 50). The questionnaire was completed correctly in 92% of cases, with only 11 participants requiring follow-up if the questionnaire was incomplete (i.e., not responding to 1 trait question/30 traits on the BSRI-30).

#### Resting hemodynamics (heart rate, blood pressure)

Resting heart rate (HR) and BP [including SBP, diastolic blood pressure (DBP), and mean arterial pressure (MAP)] were assessed using the average of the last two of three measurements collected via an automated BP assessment device (GE Dinamap ProSeries, Batesville, IN, USA). If the second and third systolic BP measurements collected were not within 5 mmHg of one another, a fourth measurement was collected with the last two averaged.

#### Arterial stiffness (pulse wave velocity)

PWV (m/s) is a measure of regional arterial stiffness, where higher values of PWV are indicative of increased arterial stiffening. PWV uses applanation tonometry to detect the pressure waveform from the skin surface of an artery using micromanometer-tipped pressure probes (SPT-301, Millar Instruments), as previously reported by our lab (20). These pressure waveforms are then band-pass filtered between 5 and 30 Hz to determine the foot of each pulse for the calculation of the pulse transit time using LabChart (AD Instruments, Colorado Springs, CO, USA). Distance between the measured locations was assessed as the average of two measurements using a measuring tape over the surface of the body. Tonometers were placed on the carotid and femoral arterial sites for the determination of central PWV, and calculated using the following formula: central PWV = (0.8 × carotid − femoral distance)/carotid − femoral pulse transit time (51). Peripheral leg PWV was calculated using waveforms at the femoral and dorsalis pedis arteries according to the following formula: leg PWV = (femoral − dorsalis pedis distance)/femoral − dorsalis pedis transit time (52, 53). Similarly, peripheral arm PWV was calculated using waveforms at the carotid and radial arteries according to the following formula: arm PWV = [radial − suprasternal notch distance − (carotid − suprasternal notch distance)]/carotid − radial pulse transit time (52, 53). Central PWV was collected in all participants (*n* = 130), but leg PWV was collected in 128 participants and arm PWV in only 82 participants, due to different collection protocols for the studies included in this project. Measurements were reported as the mean of two sets of 10 continuous heart cycles. If the PWV measurements between the two sets were not within 0.5 m/s of one another, a third set of 10 heart cycles was collected and averaged. Given the dependent of PWV on arterial blood pressure (54), measures of blood pressure were included as a covariate in statistical analysis of PWV outcomes.

#### Endothelial function and blood velocity

Participants completed a brachial artery reactive hyperemia FMD test to assess macrovascular endothelial function, where higher values of FMD are indicative of improved endothelial function. Tests were conducted in the left arm of 82 participants and in the right arm of 48 participants, based on the initial study in which participants were recruited from. Using a Doppler ultrasound machine (Vivid Q, GE Medical Systems, Horten, Norway) attached to a 12 MHz linear array probe in duplex mode with an insonation angle of 68° (55), brachial artery diameter and blood velocity was collected before cuff inflation (baseline) for 30 s. In line with current guidelines (56), a pneumatic blood pressure cuff was placed around the forearm and was rapidly inflated to suprasystolic pressure (∼200 mmHg) for 5 min to occlude blood flow to the distal artery. Arterial diameters and blood velocity were measured again using the same Doppler ultrasound machine following 4 min of cuff inflation (occlusion: 30 s) and in the 3 min immediately following cuff deflation. During the test, heart rate was collected using single-lead electrocardiogram into the Doppler ultrasound. Images were stored in a Digital Imaging and Communications in Medicine (DICOM) format, and end-diastolic frames were extracted and compiled (Sante DICOM Editor, v. 3.1.20, Santesoft, Athens, Greece, or an internally created extraction program called Pancakes). DICOM files were then analyzed using a semi-automated edge tracking software [Artery Measurement System (AMS) II, version 1.141, Gothenburg, Sweden] (57). Baseline diameter was determined as an average of the arterial diameters during rest in the 30 s prior to inflation, and peak diameter was determined as the largest five-heart cycle average of diameters in the 3 min following deflation. FMD was reported as both an absolute change (AbsFMD) and percentage change (%FMD) in diameter: FMD = peak diameter − baseline diameter; %FMD = [(peak diameter − baseline diameter)/baseline diameter] × 100%. Mean blood velocity (MBV) measures taken at the same time as ultrasound assessments, extracted as AVI files, and were analyzed using a pixel-based tracking software (Measurements from Arterial Ultrasound Imaging; Hedgehog Medical, Waterloo, ON, Canada). MBV was similarly averaged into five-heart cycle average time bins and used to calculate shear rate (SR = 8 × MBV/arterial diameter), as described previously (58). The time to peak diameter and SR areas under the curve to the time of peak diameter are reported.

#### Cardiorespiratory fitness test (V̇O_2_peak)

Participants completed an incremental exercise test to exhaustion seated upright on a stationary cycle ergometer (Lode Excalibur Sport V 2.0, Groningen, Netherlands, or Kettler Ergo Race, Kettler, Virginia Beach, VA, USA) to determine the V̇O_2_peak, in accordance with current guidelines (59). A metabolic cart with an online gas collection system (Quark CPET metabolic cart, COSMED, Italy) was used to determine oxygen consumption and carbon dioxide production. HR was monitored continuously with an HR monitor (Polar A3, Lake Success, NY, USA). The V̇O_2_peak test began with a 3-min warm-up at 50 W (or lower if the participant indicated this intensity was too intense for a warm-up), after which the power was increased by 5 W every 10 s until volitional exhaustion or the point at which pedal cadence fell below 60 rpm, as described previously (60). After reaching this point, participants continued to cycle to cool down for 2 min at 50 W or less. V̇O_2_peak (ml/kg/min) was defined as the highest oxygen consumption achieved over a 30 s period. The V̇O_2_peak test was considered successful if at least of two of the following four criteria were met: (1) perceived exertion was >17 on a Borg scale of 6–20; (2) HR was within 10 beats per minute of age-predicted maximal HR (208–0.7 × age); (3) their respiratory exchange ratio was >1.1; and (4) a plateau in V̇O_2_ was reached. If V̇O_2_peak was not achieved, the participant returned another day to perform the test again.

### Statistical analysis

All statistical analyses were performed using SPSS (version 22, IBM, Chicago, IL, USA) and R Statistical Software (v.4.1.0, R Core Team, 2023, Vienna, Austria) for allometric scaling analysis. All data were reported using descriptive statistics, including means and standard deviations for normally distributed continuous variables, medians and interquartile ranges (IQRs) for non-normally distributed continuous variables, and frequencies (percentages) for categorical variables (i.e., race, ethnicity, sex category, gender identity category, gender expression). Statistical significance was set as *p*  <0.05.

#### Primary objective (association of sex, gender identity, and gender expression with FMD and PWV)

Data were first inspected for normality using the Shapiro–Wilk test, histograms, and quantile–quantile (Q–Q) plots. If normally distributed, data were first compared between sexes using an independent *t*-test with equal variances assumed if Levene's test for equality of variances was not significant (*p* > 0.05). Independent *t*-tests that failed to meet the assumption for homogeneity of variances (*p* < 0.05) used an independent *t*-test with unequal variances assumed. If not normally distributed, Wilcoxon Rank Sum tests were conducted. Similarly, independent means for outcome data were compared between gender identities using an independent *t*-test to compare men and women, with outcomes from non-binary participants detailed but not included in the analysis due to the small sample size (*n* = 2), using the same statistical analysis methods outlined for comparisons between sexes. Some data (age, height, weight, BMI) for non-binary participants are included in ranges to remove indirect participant identifiers. The effect sizes for independent samples *t*-tests were quantified using a Cohen's *d* calculation, where a small effect is *d* = 0.2, medium effect is *d* = 0.5, and large effect is *d* > 0.8 (61). For non-parametric Wilcoxon Rank sum tests, an *r* statistic was computed (*r* = *Z*-statistic/√*n*) (62).

Then, all data were compared across gender expression categories (i.e., feminine, masculine, androgynous, undifferentiated) using a one-way ANOVA and Bonferroni-corrected *post-hoc* tests for multiple comparisons, if applicable. We inspected for homogeneity, as well as the distribution of standardized residuals. Data with high residuals were examined for potential removal. If assumptions were not met, a Kruskal–Wallis test was conducted. If differences between the four gender expressions groups were observed, a two-sample Wilcoxon Rank sum tests with Dunn–Bonferroni *post-hoc* pairwise correction tests were subsequently applied. Effect sizes were quantified using an eta-squared statistic for a one-way ANOVA, and similar eta-squared statistic based on the Kruskal–Wallis *H-*statistic, using the following equation:

$$\eta_{H}^{2}=[H-k\;\mathrm{groups}(4)+1]/(n-k)$$

A one-way analysis of covariance with Bonferroni *post-hoc* pairwise correction tests, if applicable, was used to examine the potential influences of *a priori* covariates on the main effects of sex, gender identity or gender expression: blood pressure (MAP) for central PWV, leg PWV, and arm PWV; cardiorespiratory fitness for central PWV, leg PWV, arm PWV, and %FMD; and shear rate area under the curve to peak dilation (SRAUC) for %FMD. Allometric scaling of the FMD results was performed, as supported by recent guidelines (56), if criteria were met (63, 64). Using Rtery (Github) (65), the difference between the natural logarithm of baseline artery diameter and national logarithm of peak artery diameter was entered as a dependent variable into a linear mixed-effects model that included group as an independent variable and the logarithm of baseline diameter as a covariate. The back-transformed estimated marginal means and standard deviations are reported for %FMD_scaled_.

#### Secondary objective (relationship between feminine and masculine gender expression scores with cardiorespiratory fitness, central PWV, and FMD)

Analysis of the relationship between %FMD and Central PWV (m/s) and BSRI-Feminine score, BSRI-Masculine score, and cardiorespiratory fitness (V̇O_2_peak, ml/kg/min) were analyzed using a Pearson correlation. Furthermore, an analysis of the relationship between baseline diameter and %FMD was conducted using a Pearson correlation. To assess or internal consistency of the BSRI within this population, Cronbach's alpha calculation was performed to examine the relationship between the 10 questions within each BSRI subcategory (masculine, feminine, neutral), with a score of 1 indicating perfect correlation and 0 indicating no correlation between items; in general, a score between 0.7 and 0.95 was indicative of acceptable scores for internal consistency (66–68).

## Results

### Participant characteristics

**Figure 1:**
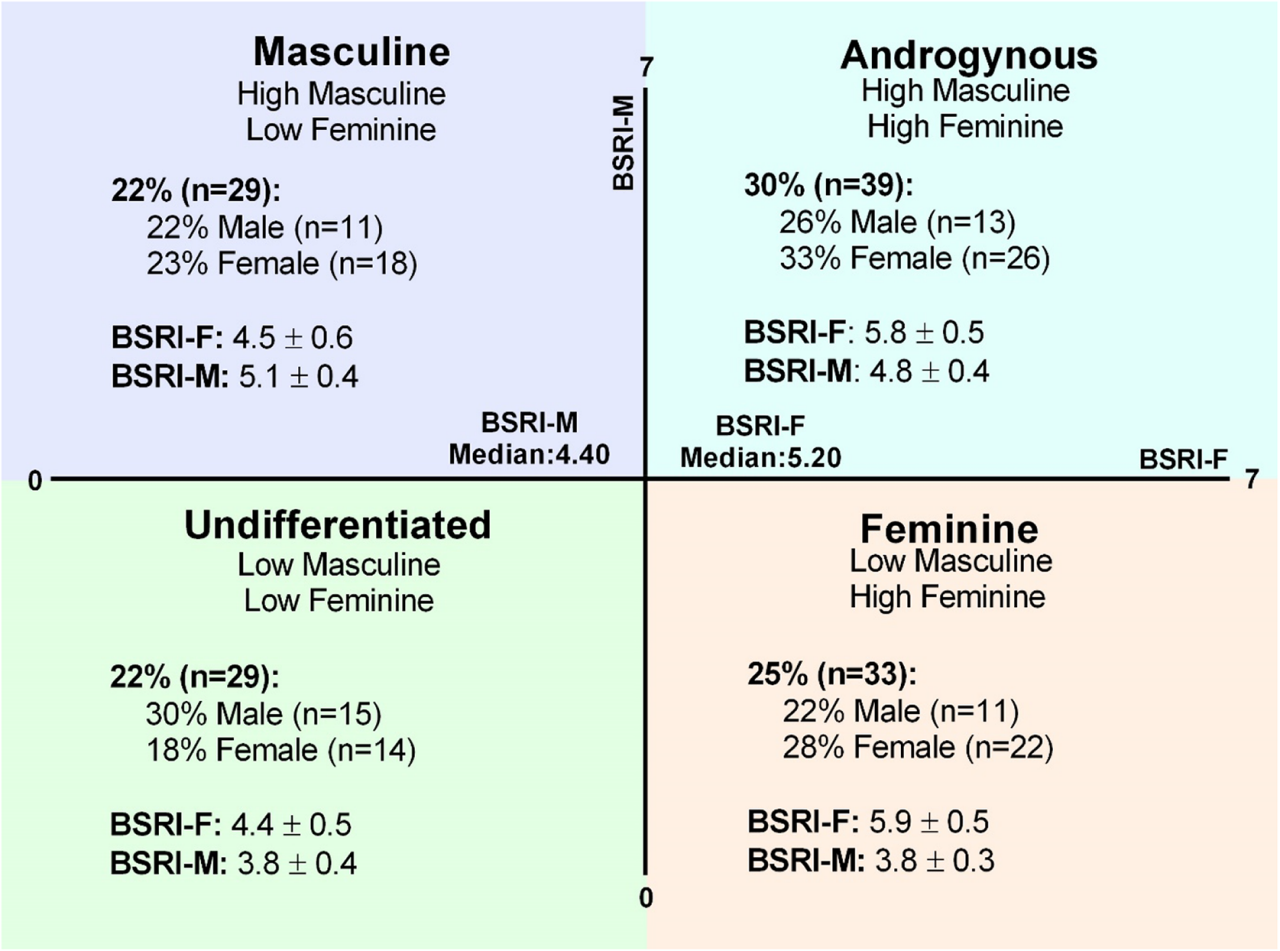
Gender expression groups. The BSRI creates gender scores for the feminine (BSRI-F) and masculine (BSRI-M) subscales that can be used to generate median-split quadrants for masculine, androgynous, undifferentiated, and feminine gender expression groups. The proportion of the overall sample in each category and the prevalence of males and females in each gender expression group are shown in detail. Mean scores for the BSRI-F and BSRI-M are represented (mean ± SD) in each gender expression category.

Of the 130 participants recruited, 50 identified as male (38%) while 80 identified as female (62%). Gender identity groups included 49 men (38%), 79 women (61%), and 2 non-binary participants (2%). The proportion of participants categorized into gender expression groups is outlined in Figure 1. Ethnicity and race of the participants are detailed in full in Supplementary Table S2. Briefly, the ethnicity of the participants included in this study is as follows: African Origins (*n* = 5; 4%), Asian Origins (*n* = 54; 42%), European Origins (*n* = 38; 29%), Mixed Origins (*n* = 30; 23%), and North American Origins (*n* = 3; 2%). The race of the participants included in this study is as follows: Asian (*n* = 48; 37%), Black or African American (*n* = 2; 2%), Middle Eastern or North African (*n* = 10; 8%), Mixed (*n* = 8; 6%), Prefer to Self-Describe (*n* = 2; 2%), and White or Caucasian (*n* = 60; 46%).

While the average age of the participants across sex and gender identity category groups were not different, all other characteristics measured were higher in males compared to females, and men compared to women, including height, weight, BMI, and V̇O_2_peak (all *p* < 0.01; Table 1). There was no difference across gender expression groups for any participant characteristics (Table 2).

**Table 1:** Participant characteristics, resting hemodynamics, and flow-mediated dilation test outcomes characterized by sex and gender identity categories.

	Males (*n* = 50)	Females (*n* = 80)		Men (*n* = 49)	Women (*n* = 79)	Non-binary (*n* = 2)	*p*-values	Effect sizes
Age (years)	22 [4]	21 [4]		22 [4]	21 [4]	Both 18–25	S: *p* = 0.31 G: *p* = 0.24	S: *r* = 0.09 G: *r* = 0.10
Height (cm)	178 ± 7 (176–180)	164 ± 7^a^ (163–166)		178 ± 7 (176–180)	164 ± 7^b^ (163–166)	160–170 175–185	**S: *p* < 0.01** **G: *p* < 0.01**	S: *d* = 2.10 G: *d* = 2.08
Weight (kg)	81.4 [18.9]	60.8 [13.1]^a^		80.9 [18.3]	60.7 [12.0]^b^	95–100 90–95	**S: *p* < 0.01** **G: *p* < 0.01**	S: *r* = 0.60 G: *r* = 0.62
BMI (kg/m^2^)	25.0 [5.9]	22.7 [4.1]^a^		25.0 [5.8]	22.4 [4.1]^b^	35–40 25–30	**S: *p* < 0.01** **G: *p* < 0.01**	S: *r* = 0.31 G: *r* = 0.32
V̇O_2_peak (mL/kg/min)	44.2 ± 8.9 (41.6–46.7)	39.1 ± 8.0^a^ (37.4–40.9)		44.4 ± 8.9 (41.9–46.9)	39.3 ± 7.9^b^ (37.5–41.1)	27.5, 32.4	**S: *p* < 0.01** **G: *p* < 0.01**	S: *d* = 0.60 G: *d* = 0.62
SBP (mmHg)	117 ± 8 (115–120)	106 ± 7^a^ (104–107)		117 ± 8 (115–119)	106 ± 7^b^ (104–107)	110, 138	**S: *p* < 0.01** **G: *p* < 0.01**	S: *d* = 1.58 G: *d* = 1.57
DBP (mmHg)	63 [7]	63 [9]		62 [7]	63 [10]	57, 72	S: *p* = 0.54 G: *p* = 0.71	S: *r* = 0.05 G: *r* = 0.03
MAP (mmHg)	84 ± 6 (82–86)	79 ± 6^a^ (77–80)		84 ± 6 (82–86)	79 ± 6^b^ (77–81)	78, 97	**S: *p* < 0.01** **G: *p* < 0.01**	S: *d* = 0.87 G: *d* = 0.83
HR (bpm)	64 ± 10 (61–67)	62 ± 9 (60–64)		64 ± 9 (61–66)	62 ± 9 (60–64)	66, 92	S: *p* = 0.20 G: *p* = 0.32	S: *d* = 0.23 G: *d* = 0.18
Baseline diameter (mm)	4.03 [0.63]	3.19 [0.68]^a^		4.04 [0.67]	3.19 [0.65]^b^	3.86, 3.94	**S: *p* < 0.01** **G: *p* < 0.01**	S: *r* = 0.63 G: *r* = 0.63
Peak diameter (mm)	4.38 [0.61]	3.53 [0.61]^a^		4.38 [0.61]	3.53 [0.61]^b^	4.04, 4.02	**S: *p* < 0.01** **G: *p* < 0.01**	S: *r* = 0.65 G: *r* = 0.65
AbsFMD (mm)	0.27 ± 0.12 (0.24–0.30)	0.27 ± 0.11 (0.25–0.30)		0.27 ± 0.12 (0.24–0.31)	0.27 ± 0.11 (0.25–0.30)	0.18, 0.09	S: *p* = 0.84 G: *p* = 0.94	S: *d* = 0.04 G: *d* = 0.01
Baseline MBV (cm/s)	8.0 [5.9]	6.5 [3.0]^a^		8.0 [6.1]	6.5 [3.0]^b^	6.1, 7.1	**S: *p* = 0.01** **G: *p* = 0.02**	S: *r* = 0.22 G: *r* = 0.22
Baseline SR (s^−1^)	163.2 [138.7]	161.3 [70.9]		163.3 [139.1]	161.5 [72.4]	127.3, 144.0	S: *p* = 0.91 G: *p* = 0.92	S: *r* = 0.01 G: *r* = 0.01
SRAUC (×10^3^ s^−1^)	1.68 [1.90]	2.03 [2.14]		1.64 [1.83]	2.10 [2.15]	7.1, 37.4	S: *p* = 0.53 G: *p* = 0.38	S: *r* = 0.05 G: *r* = 0.08
Time to peak diameter (s)	46 [21]	42 [19]		46 [20]	42 [19]	29, 57	S: *p* = 0.46 G: *p* = 0.61	S: r = 0.06 G: *r* = 0.04

S, sex; G, gender identity; BMI, body mass index; V̇O_2_peak, volume of oxygen at peak exercise capacity; SBP, systolic blood pressure; DBP, diastolic blood pressure; MAP, mean arterial pressure; HR, heart rate; AbsFMD, absolute flow-mediated dilation response; MBV, mean blood velocity; SR, shear rate; SRAUC, shear rate area under the curve to peak dilation; d, Cohen's *d* value to denote effect size for independent *t*-test; *r*, effect size for non-parametric Wilcoxon Rank Sum test.

Independent *t*-tests were performed comparing between sexes (male/female) and gender identity category groups (men/women). Non-binary participants were characterized detailing the outcome values from each participant (*n* = 2), but not included in statistical analysis due to low sample size, with age, height, weight, and BMI included as ranges to avoid indirect participant identifiers. Significant main effects are bolded. Normally distributed outcomes are represented as mean ± standard deviation (SD) (95% CI), while non-normally distributed outcomes are represented as median [IQR].

^a^ Significantly higher in males compared to females.

^b^ Significantly higher in men compared to women.

**Table 2:** Participant characteristics and resting hemodynamics characterized by gender expression.

	Masculine (*n* = 29)	Feminine (*n* = 33)	Androgynous (*n* = 39)	Undifferentiated (*n* = 29)	*p*-values	Effect sizes (*η*^2^)
Age (years)	20 [4]	22 [4]	20 [3]	22 [6]	0.12	$\eta_{H}^{2}$ = 0.02
Height (cm)	168 ± 8 (165–171)	169 ± 12 (165–174)	169 ± 9 (166–172)	173 ± 8 (170–176)	0.21	*η*^2^ = 0.04
Weight (kg)	65.9 [17.8]	64.2 [24.2]	63.9 [21.4]	71.3 [25.5]	0.54	$\eta_{H}^{2}$ = 0.01
BMI (kg/m^2^)	24.6 [5.3]	23.2 [3.5]	23.5 [4.8]	23.7 [5.0]	0.70	$\eta_{H}^{2}$ = 0.01
V̇O_2_peak (ml/kg/min)	41.3 ± 8.1 (38.2–44.40)	39.6 ± 8.0 (36.7–42.5)	41.5 ± 8.6 (38.7–44.3)	42.0 ± 10.1 (38.1–45.8)	0.72	*η*^2^ = 0.01
SBP (mmHg)	112 ± 10 (109–116)	109 ± 11 (105–112)	109 ± 8 (107–112)	111 ± 10 (107–115)	0.36	*η*^2^ = 0.03
DBP (mmHg)	62 [9]	62 [9]	63 [8]	63 [7]	0.66	$\eta_{H}^{2}$ = 0.01
MAP (mmHg)	81 ± 6 (79–84)	81 ± 8 (78–84)	80 ± 6 (78–82)	82 ± 6 (79–84)	0.83	*η*^2^ = 0.01
HR (bpm)	62 ± 9 (59–65)	63 ± 7 (60–65)	63 ± 9 (60–66)	64 ± 12 (60–69)	0.81	*η*^2^ = 0.01
Baseline diameter (mm)	3.85 [1.26]	3.39 [0.84]	3.44 [0.90]	3.65 [0.67]	0.37	$\eta_{H}^{2}$ < 0.01
Peak diameter (mm)	4.09 [1.16]	3.61 [0.76]	3.77 [0.93]	3.91 [0.90]	0.40	$\eta_{H}^{2}$ < 0.01
AbsFMD (mm)	0.27 ± 0.13 (0.22–0.32)	0.30 ± 0.11 (0.26–0.34)	0.27 ± 0.10 (0.23–0.30)	0.25 ± 0.11 (0.21–0.29)	0.36	*η*^2^ = 0.03
Baseline MBV (cm/s)	7.4 [5.3]	6.8 [2.9]	7.0 [5.9]	6.6 [3.5]	0.60	$\eta_{H}^{2}$ = 0.01
Baseline SR (s^−1^)	166.8 [134.1]	163.2 [70.6]	174.8 [146.6]	144.0 [78.4]	0.24	$\eta_{H}^{2}$ = 0.01
SRAUC (×10^3^ s^−1^)	2.24 [2.89]	2.10 [2.43]	1.82 [1.68]	1.73 [1.87]	0.56	$\eta_{H}^{2}$ = 0.01
Time to peak diameter (s)	48 [22]	49 [19]	41 [18]	40 [21]	0.17	$\eta_{H}^{2}$ = 0.02

BMI, body mass index; V̇O_2_peak, volume of oxygen at peak exercise capacity; SBP, systolic blood pressure; DBP, diastolic blood pressure; MAP, mean arterial pressure; HR, heart rate; AbsFMD, absolute flow-mediated dilation response; MBV, mean blood velocity; SR, shear rate; SRAUC, shear rate area under the curve to peak dilation; *η*^2^, eta-squared value to denote effect size for a one-way ANOVA; $\eta_{H}^{2}$, eta-squared valued based on the *H*-statistic from the Kruskal–Wallis non-parametric test.

One-way ANOVAs were performed comparing across gender expression groups. Normally distributed outcomes are represented as mean ± standard deviation (SD) (95% CI), while non-normally distributed outcomes are represented as median [IQR].

### Bem Sex Role Inventory Scores

BSRI-Feminine scores were higher on average in females compared to males [female: 5.4 ± 0.8, 95% confidence interval (CI): 5.2–5.8; male: 5.0 ± 0.9, 95% CI: 4.7–5.2; *p* < 0.01, *r* = 0.50], and also higher in women compared to men (women: 5.4 ± 0.8, 95% CI: 5.2–5.6; men: 5.0 ± 0.9, 95% CI: 4.7–5.2; *p* = 0.01, *r* = 0.47; non-binary: 5.4 and 3.7). In contrast, there was no difference for the BSRI-Masculine scores across sex categories (female: 4.4 ± 0.7, 95% CI: 4.2–4.5; male: 4.4 ± 0.7, 95% CI: 4.2–4.5, *p* = 0.85, *r* = 0.03) or gender identity category groups (women: 4.4 ± 0.7, 95% CI: 4.2–4.5l men: 4.4 ± 0.7, 95% CI: 4.2–4.6, *p* = 0.93, *r* = 0.02; non-binary: 4.4 and 3.8).

BSRI-Feminine scores were higher in feminine (5.9 ± 0.5) and androgynous (5.8 ± 0.5) gender expression groups, compared to masculine (4.5 ± 0.6) and undifferentiated (4.4 ± 0.5) gender expression groups (*p* < 0.01 for all, *η*^2^ = 0.65), with no difference between the feminine and androgynous (*p* = 1.00) and the masculine and undifferentiated (*p* = 1.00) gender expression groups (Figure 1). BSRI-Masculine scores were lower in the feminine (3.8 ± 0.3) and undifferentiated (3.8 ± 0.4) groups compared to the masculine (5.1 ± 0.4) and androgynous (4.8 ± 0.4) gender expression groups (*p* < 0.01 for all, *η*^2^ = 0.71), with no difference between the feminine and undifferentiated groups (*p* = 1.00; Figure 1). However, BSRI-Masculine was also higher in the masculine compared to the androgynous gender expression group (*p* = 0.03; Figure 1).

To assess the internal consistency of each subscale (feminine, masculine, neutral) of the BSRI, Cronbach's alphas were computed: BSRI-Feminine *α* = 0.87, BSRI-Masculine *α* = 0.75, and BSRI-Neutral *α* = 0.40; BSRI-Feminine and BSRI-Masculine are within acceptable scores for internal consistency (66–68).

### Association of sex and gender with resting hemodynamics

Resting SBP and MAP were higher on average in males compared to females (*d* = 1.58 and 0.87 respectively; both *p* < 0.01; Table 1) and higher in men compared to women (*d* = 1.57 and 0.83 respectively; both *p* < 0.01; Table 1). There were no sex or gender identity category differences for DBP or HR (Table 1). There were no differences across gender expression groups for any hemodynamic measure (SBP, DBP, MAP, and HR; Table 2).

### Primary objective: association of sex and gender with arterial stiffness (PWV)

**Figure 2:**
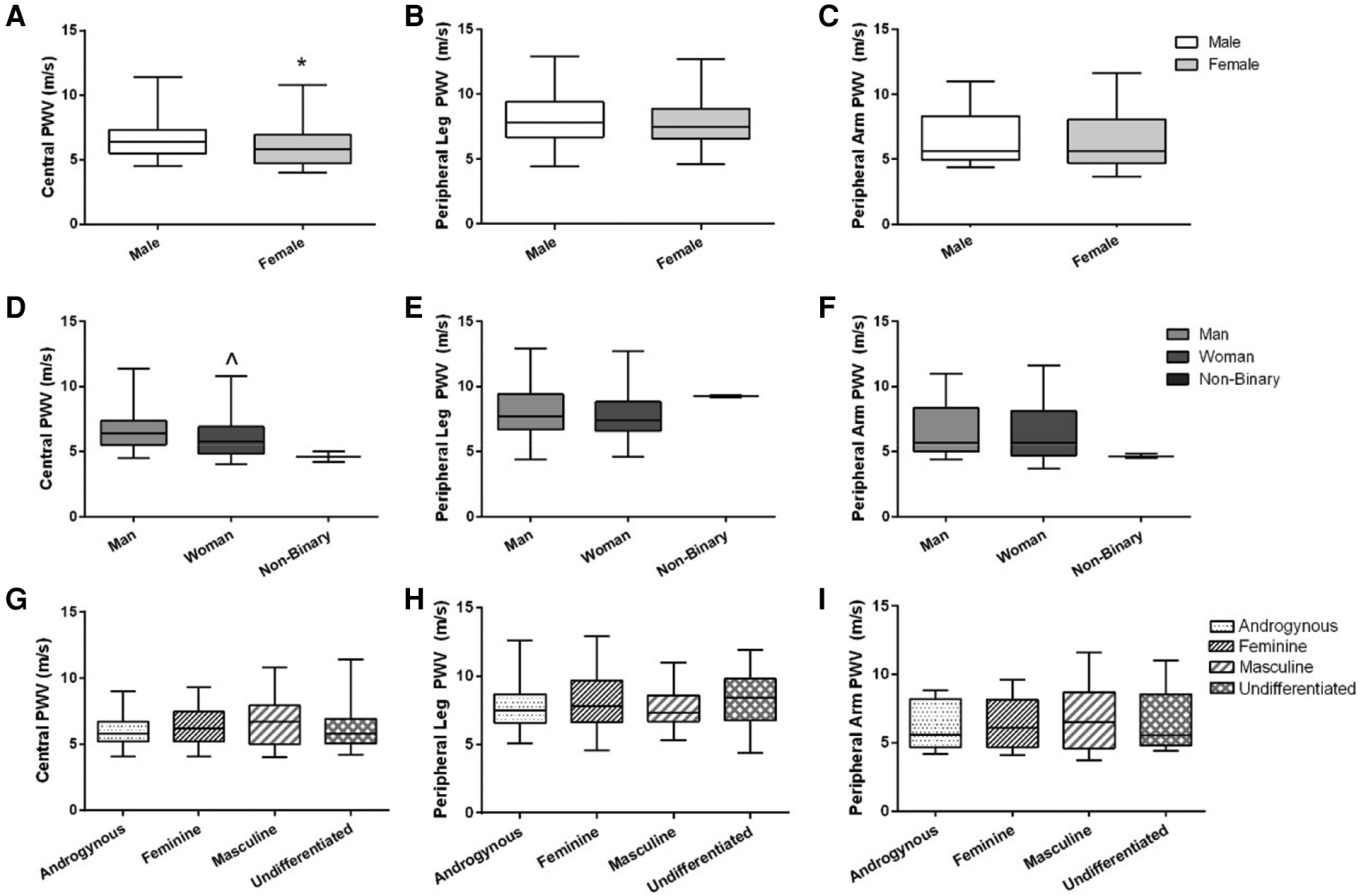
Arterial stiffness (central, peripheral leg, peripheral arm) across sex, gender identity, and gender expression groups. Data are illustrated in **(A–C)** for sex, **(D–F)** for gender identity groups (non-binary data are illustrated on the graphs but not included in the statistical analysis), and **(G–I)** for gender expression groups. Box-and-whisker plots represent the median: the box represents the interquartile range, with the minimum and maximum points represented by the whiskers. Independent *t*-tests were performed between males and females and men and women. A one-way ANOVA was performed to examine %FMD across gender expression groups. *Females have lower central PWV compared to males (*p* = 0.02). ^Women have lower central PWV compared to men (*p* = 0.02). There were no differences between sexes or gender identity category groups for peripheral leg PWV or peripheral arm PWV, or across gender expression groups for any PWV outcome.

Central PWV was higher on average in males compared to females [6.4 (1.8) vs. 5.8 (2.2) m/s, *p* = 0.02; *r* = 0.20; Figure 2A], and higher in men compared to women [6.4 (1.9) vs. 5.8 (2.1) m/s, *p* = 0.02; *r* = 0.20; Figure 2D]. There were no differences in peripheral leg PWV (Figures 2B,E) or peripheral arm PWV (Figures 2C,F) between sex or gender identity category groups. Similarly, there were no differences across gender expression groups for any PWV measures (Figures 2G–I). None of these findings were altered when the independent association of MAP or V̇O_2_peak was added as covariates.

### Primary objective: association of sex and gender with endothelial function (FMD)

**Figure 3:**
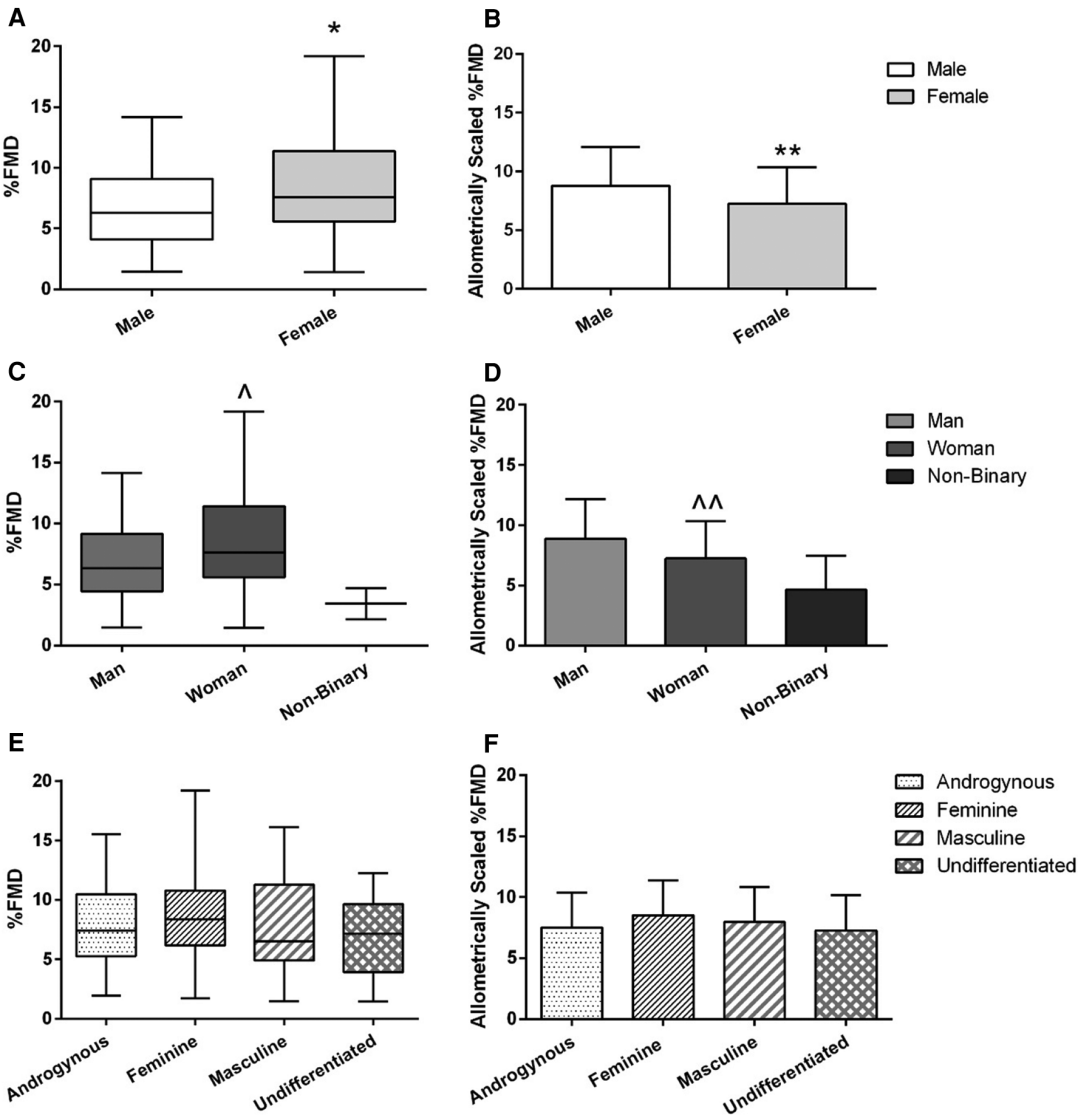
FMD across sex **(A,B)**, gender identity **(C,D)**, and gender expression **(E,F)** groups. Left-side graphs are all %FMD, unscaled for baseline diameter **(A,C,E)**; right-side graphs are all %FMD allometrically scaled to baseline diameter **(B,D,F)**. Graphs for %FMD (unscaled) show mean ± SD with individual data points, while %FMD (scaled) show no data points. Independent *t*-tests were performed between males and females and men and women. A one-way ANOVA was performed to examine %FMD across gender expression groups. *Females have higher %FMD (without controlling for baseline diameter differences) compared to males (*p* = 0.01). **Females have lower %FMD when controlling for baseline diameter using allometric scaling (*p* = 0.03). ^Women have higher %FMD (without controlling for baseline diameter differences compared to men (*p* = 0.02). ^^Women have lower %FMD when controlling for baseline diameter using allometric scaling (*p* = 0.03).

Baseline brachial artery diameter and peak brachial artery diameter were both larger on average in males compared to females (*p* < 0.01 both; *r* = 0.63 and 0.65 respectively; Table 1) and men compared to women (*p* < 0.01 both; *r* = 0.63 and 0.65 respectively; Table 1). AbsFMD was not different between sex or gender identity category groups (Table 1). %FMD was initially higher on average in females compared to males (8.5 ± 3.7 vs. 6.9 ± 3.5%; *p* = 0.01, *d* = 0.45; Figure 3A) and in women compared to men (8.6 ± 3.7 vs. 7.0 ± 3.5%; *p* = 0.02, *d* = 0.44; Figure 3C); this result remained significant when V̇O_2_peak or SRAUC were added as covariates in the model (both *p* = 0.02). After allometric scaling to consider differences in artery size, %FMD_scaled_ was higher on average in males compared to females (8.8 ± 3.3 vs. 7.2 ± 3.1%, *p* = 0.03, *r* = 0.42; Figure 3B) and in men compared to women (8.9 ± 3.3 vs. 7.2 ± 3.1, *p* = 0.02, *r* = 0.47; Figure 3D).

Baseline MBV was higher on average in males compared to females (*p* = 0.01, *r* = 0.22; Table 1) and men compared to women (*p* = 0.02, *r* = 0.22; Table 1). There were no sex or gender identity category group differences for baseline SR, SRAUC, or time to peak diameter (Table 1). There was no difference in any endothelial function or blood velocity outcome across gender expression groups (Table 2; Figure 3E); this result remained non-significant when %FMD was allometrically scaled (*p* = 0.39; Figure 3F) or when V̇O_2_peak or SRAUC was considered as a covariate.

### Non-binary participant outcomes

Due to a low sample size for gender-diverse participants, non-binary participants were qualitatively, instead of statistically, examined. Compared to men and women, non-binary participants may have higher BMI (25–30 and 35–40 kg/m^2^) and lower cardiorespiratory fitness (27.5 and 32.4 ml/kg/min; Table 1). Non-binary participants also may have elevated blood pressure (SBP: 110 and 138, MAP: 78 and 97 mmHg; Table 1) and resting heart rate (66 and 92 bpm; Table 1) compared to women, impaired endothelial function (AbsFMD: 0.18 and 0.09 mm; %FMD: 4.69% and 2.16%, Table 1 and Figures 3C,D), including after differences in artery size were considered using allometric scaling (%FMD_scaled_: 4.7 ± 2.8%). However, non-binary participants had lower central (4.2 and 5.3 m/s) and peripheral arm PWV (4.8 and 4.5) but elevated peripheral leg PWV (9.2 and 9.3 m/s).

### Secondary objective: relationship between gender expression, cardiorespiratory fitness and endothelial function, central PWV

There was no significant relationship between gender expression scores (BSRI-Feminine, BSRI-Masculine) and endothelial function or central PWV (Supplementary Figures S1A–D). Similarly, there was no relationship between cardiorespiratory fitness and endothelial function (Supplementary Figure S1E) or central PWV (Supplementary Figure S1F). Finally, there was a negative relationship between baseline diameter and %FMD (*r* = −0.55, *p* < 0.001; Supplementary Figure S1G).

## Discussion

This is the first study to investigate the associations between sex, gender identity, and gender expression and novel and traditional risk factors for CVD in a young, healthy population. We found that males had higher blood pressure (SBP, MAP; large effect) and central arterial stiffness (central PWV; small effect) compared to females in both unadjusted and adjusted models. However, males had a greater %FMD compared to females (medium effect), when larger artery diameters in males were considered using allometric scaling. Given most of the participants (∼99%) were cisgender, the results comparing gender identity categories (men/women) were not different for most sex categories, and therefore potential differences between sex and gender identity categories cannot be fully elucidated. However, there were no differences across gender expression groups (i.e., feminine, masculine, androgynous, undifferentiated) for any outcome. Finally, there was no relationship between gender expression scores (BSRI-F and BSRI-M) or cardiorespiratory fitness and either central PWV or %FMD. These results suggest that in healthy, young cisgender adults, sex and gender identity category influence several cardiovascular risk factors, but gender expression does not appear to influence these outcomes. Due to the small population of gender-diverse participants, examination of non-binary, transgender, and other gender-diverse groups was not possible in this study and warrants further investigation in future studies with great sample sizes of gender-diverse individuals.

### Association of sex with hemodynamics and arterial stiffness

The present study found that males had elevated blood pressure (SBP and MAP) compared to females, with no difference in DBP or HR. Using an electronic tool (sexdifference.org) to estimate the overlap between the normal distributions in males and females, we also observed that the overlap between sexes was ∼46% for SBP and ∼68% for MAP (69). Taken alongside the large effect sizes for SBP (*d* = 1.58) and MAP (*d* = 0.87), sex differences in BP are evident. These findings are in line with most previous research on sex differences in hemodynamics, finding higher SBP, MAP, and occasionally DBP in males compared to females (20, 21, 36, 70–74). Research by Harris et al. found that males had higher SBP compared to females (70). Similarly, research in our lab also found elevated SBP and MAP in males compared to two groups of premenopausal females: natural cycling and combined oral contraceptive pill users (20) Finally, a recent narrative review discussed that resting blood pressure is elevated in males compared to females and that this may be a critical factor in the increased risk for CVD in males earlier in life than females (36). The mechanisms underlying elevated BP in males compared to females include sex hormones that may involve testosterone increasing blood pressure and 17β-estradiol decreasing blood pressure (and the ratio of testosterone/estradiol) (75–78), and anatomical differences in height resulting in increased pulse wave propagation that must be accompanied by a heightened SBP in males (18). While sex hormones were not measured in this study, males were taller than females, which could be in part responsible for these differences in blood pressure. In addition, sex and gender factors are challenging to separate, and gender-related lifestyle factors [i.e., smoking, alcohol consumption, sodium intake, sleep (79)] may also play a role influencing observed sex differences in blood pressure. However, the finding that BP is higher in males compared to females is not always true; BP is reported to be the same or elevated in females compared to males in populations with elevated CVD risk factors, including elevated BMI and low fitness levels (22, 80). Therefore, it is plausible that the “female advantage” observed with blood pressure previously may be outweighed by the influence of additional risk factors for CVD, such as obesity and low fitness.

In the present study, males had elevated central PWV, but not peripheral arm or leg PWV compared to females, which was not explained by controlling for blood pressure elevations in males. Again, using an electronic tool detailed above (69), the overlap between sexes for central PWV was ∼86% with a small effect size (*r* = 0.20), suggesting that while sex differences in central PWV were significant, the magnitude of difference was more subtle than BP. This is in line with some (20–22, 36, 71), but not all (73), studies that have observed sex differences in local (carotid artery) and/or systemic arterial stiffness. For example, research by Baldo et al. found that central PWV was higher in males compared to females, across the aging lifespan (21). The same was true in a population of patients with pre-hypertension to stage 1 hypertension, with males having higher central PWV than females, despite females having a higher SBP compared to males (22). Marlatt et al. identified that sex differences in carotid artery compliance became present in early adulthood (∼late 30s), but not in childhood (71). This study, alongside previous research, points to the role of sex hormones like 17β-estradiol to play a role in these apparent sex differences and/or the influence of sex differences in growth patterns between males and females (17).

Similarly, recent work found that β-stiffness of the carotid artery was higher in males compared to females who were naturally cycling or used oral contraceptive pills (20). Despite finding a sex difference in local arterial stiffness in the carotid artery, no differences were observed in central PWV, although it is possible that they were underpowered to detect a sex difference in central PWV (20). Our study, with 130 participants found a significant sex difference in central PWV between males and females (*p* = 0.02), but arguably the ∼0.6 m/s difference may not be clinically significant. Prior research has determined that a 1 m/s increase in arterial stiffness is associated with a 15% (95% CI: 9%–21%) increased risk for CVD mortality (81). While a 0.6 m/s difference in central PWV may still increase the risk for CVD marginally, it is unlikely to be solely responsible for differential rates in CVD in males and females. Furthermore, the small effect size and substantial overlap in normal distribution curves between sexes suggests that sex differences in central PWV are subtle.

### Association of sex and endothelial function

We found that unscaled %FMD was elevated in female compared to male participants but %FMD may have been artificially inflated given that baseline brachial artery diameter was also smaller in females. After performing allometric scaling analysis to account for differences in baseline artery diameter, we found that scaled %FMD was elevated in males compared to females. Using an electronic tool detailed above (69), the overlap between sexes for %FMD_scaled_ was ∼80% with a medium effect size (*d* = 0.45), suggesting that while sex differences in %FMD_scaled_ were significant the magnitude of difference between sexes was only moderately pronounced. These findings are aligned with most prior %FMD (unscaled) research finding greater %FMD in females (12, 14, 70, 82, 83), and prior research from our lab finding %FMD (scaled) is greater in males (14).

The present study also aligns with a consistent finding that male arteries are, on average, larger than female arteries (12, 70, 80, 82–84). Early research identified sex differences in reductions in %FMD with aging, alongside sex differences in %FMD and in artery diameter (85). Furthermore, this study reported a negative relationship between %FMD and resting arterial diameter, which has been extensively replicated in healthy and clinical populations (12, 83, 85). Recent research by Holder et al. observed marked sex differences in %FMD (higher in females), developing reference ranges in a large population of both healthy and clinical participants (12), following FMD guidelines (12, 56). We observed similar %FMD values compared to the reference intervals (12): present study data in the ∼50th percentile of reported references: male baseline diameter: 4.09 ± 0.49 mm; male %FMD: 6.86 ± 3.50%; female baseline diameter: 3.30 ± 0.46 mm, female %FMD: 8.51 ± 3.70%.

Age and sex differences in %FMD relate to differences in baseline diameter and further allude to structural influences on artery function between sexes (12). One evident reason for sex differences in artery diameter is a positive relationship between baseline diameter and height. Recent work by this same group also found that a “…10 cm increase in height is associated with a 0.16 mm increase in baseline diameter and a 0.28% decrease in FMD…” and this finding was independent of sex (12). In our study, there was a 14 ± 7 cm difference in height between males and females, which would have been attributed to an ∼0.23 mm increase in baseline diameter and an ∼0.39% decrease in %FMD. While sex differences in height do not fully explain the sex differences in baseline diameter and %FMD in the present study, artery size differences cannot be ignored in examining sex differences in endothelial function through allometric scaling of %FMD to baseline diameter. Overall, while unscaled %FMD may initially suggest that females have improved endothelial function compared to males, accounting for artery size differences result in males having elevated %FMD (scaled) compared to females. Researchers should consider using allometric scaling when comparing between sexes to consider baseline differences in artery size and ensure valid interpretation of sex difference findings.

### Association of gender and CVD risk factors

While there is evidence of alterations in CVD and CVD risk factors associated with gender and gender-related factors, the present study did not observe any variation in novel and traditional CVD risk factors across gender expression groups. Prior research has observed the association between gender or gender-related factors and CVD; for example, research by Pelletier et al. found that recurrent acute coronary syndrome was associated with “femininity” as a composite score of gender roles and expression (assessed by the BSRI) in middle-aged (aged ∼48 years) individuals, after adjusting for sex (34). Similarly, previous research has found that gender-related roles including caregiver burden (29), role strain (e.g., workplace-home life role stress), and other psychosocial stressors are predominant in women compared to men and attributed to increased risk factors and development of CVD (30–32). The present study did not see impaired cardiovascular health (i.e., blunted %FMD or increased PWV) in young individuals (aged ∼22 years) with higher femininity (gender expression) scores; however, it is possible that gender expression and other gender-related factors such as gender roles may not manifest until later in life. For example, caregiving burden may not be present until middle- and older-adulthood where parenting roles and care for aging relatives commonly occurs (86); therefore, any influence of these gender-related factors may not have yet progressed to the stage of negative remodeling in the vasculature. These factors may also intersect with known increases in CVD risk associated with age (9), though further research is necessary. It is also possible that gender roles have a stronger relationship with CVD health outcomes and that gender expression is less associated; further research investigating these constructs is needed in young adults. In addition, given the limitations of the BSRI in only examining gender expression, further examining of gender roles in professional and home life, along with exposure to gendered expression and roles in family and friend circles, may have added further depth to this analysis. A newly developed questionnaire by Nielsen et al., examining seven gender-related variables across domains of gender norms, gender-related traits (gender expression), and gender relations in American undergraduate students and younger adults, may be useful in extending this research (87).

In examining gender identity category groups, we observed the same sex differences detailed above in gender identity groups of men and women. The finding that ∼99% of participant sex aligned with their gender identity (male = man, female = woman) substantiates this overlap. Considering that sex and gender identity category are highly interrelated, the same conclusions detailed for sex may also be attributed to gender identity differences; furthermore, there may be gender identity influences on seemingly sex differences. As a result, separating categorical sex assigned at birth and biological factors from gender identity and sociocultural factors is not possible given this overlap in this study. However, while sex differences and gender identity differences aligned in this study, these are two different identity constructs and should be represented separately, especially to allow for the representation of gender-diverse participants.

In this study, we recruited a small number of gender-diverse individuals (*n* = 2 non-binary; comprising ∼1.5% of the total study population). While this is not representative of the diverse groups of gender queer participants that could have been recruited (i.e., transgender, genderqueer, two-spirit, etc.), this number of participants is proportionally representative of the number of gender-diverse individuals on average. For example, in Canada, 0.13% of the population report being non-binary (88), though this may be an underestimation. When considering gender-diverse individuals in analyzing for differences in cardiovascular outcomes across gender identity groups, we removed non-binary individuals from the analysis due to this low sample size and instead reported the data qualitatively in hopes of stimulating further needed research in this population. Qualitatively, it appears that the two non-binary participants in this study may have some elevated risk factors for CVD, including elevated blood pressure (above 90th percentile for SBP in the present study), impaired endothelial function (below 15th percentile for %FMD in the present study), and elevated leg PWV (above 75th percentile in the present study), though lower central and arm PWV. This could be in part due to higher BMI and lower cardiorespiratory fitness in these participants. While these findings are limited and must be explored further with a larger sample size, they do align with current literature indicating increased CVD risk factors in gender-diverse populations (89, 90), in part attributed to gender minority stress (89, 91) and increased allostatic load, or the accumulation of stress and life events, in some gender and sexual minority groups (92).

Overall, while gender expression differences were not observed in this study, this does not mean that differences do not exist or should not be studied by researchers. On the contrary, given that this study was in a young, healthy population, we may have yet to observe CVD risk factor elevations or disease manifestation later in the time course of CVD development. Similarly, it is possible that cardiovascular risk may only be apparent during periods in which gender expression or roles are challenged. For example, research by Kramer et al. found that men presented with low masculinity feedback experienced an exaggerated vagal withdrawal response during a speech task compared to those who received higher feedback (93). Overall, it is critical for researchers to continue to examine gender identity, expression, and gender-related factors, alongside sex and sex-related factors, and how they influence novel and traditional CVD risk factors.

### Limitations

While this study had several strengths, including its inclusion of several novel and traditional CVD risk factor outcomes, recruitment of participants with a wide range of cardiorespiratory fitness levels, compilation of outcomes collected using the same standardized methodologies, and a reasonably large sample size, there are some limitations to consider. First, the findings of this study are only generalizable to young, healthy adults who were primarily university students, and where the majority had alignment between their sex and gender identity. As a result, further research is necessary, particularly in middle-aged and older adults when gender expression changes alongside critical gender milestones and other contextual factors (94–96). Similarly, though representative of the proportions of gender-diverse populations in Canada, the number of non-binary participants was too low to make conclusions; further research is needed in this population adequately powered to draw conclusions about CV health. Second, the gender assessment in this study was limited to gender identity and expression, measured by the BSRI. While other gender assessment tools exist (48, 87), their utility in a university population is challenged as many questions ask about gender-related roles in familial structures, financial status in a family or income, caregiver strain, workplace role and environment, among others, many of which are less applicable in a university student context. The BSRI, in contrast, was created using data from a university population and has applicability in this context (45), albeit a limited assessment of gender expression. However, the BSRI also may use some outdated gender stereotypical traits to explore gender expression, as it was constructed in the 1970s and gender norms have shifted since (28, 97–99); though at the time of the study's design, it was the only validated and widely used gender scoring system available recommended by experts (25). Further research is necessary to create additional relevant gender assessment methods in young adults. Another limitation of the questionnaire was that a small number of participants (*n* = 11) required follow-up prompting to complete one question on the BSRI-30; this is unlikely to alter the results of the study given the lower number of participants (∼8%) requiring follow-up and the high internal consistency scores of the BSRI-30 questionnaire. Finally, the type of contraceptive or hormonal cycle phase was not controlled for in this study. Previous research has found conflicting results on the influence of contraceptives and the hormonal cycle on endothelial function and arterial stiffness (14, 52, 70, 100–103). However, recent work from our lab and others have observed no impact of contraceptives, contraceptive cycle or menstrual cycle on arterial stiffness (20), and a small influence of the menstrual cycle on endothelial function (104). Therefore, any effects of contraceptive type or hormonal cycle phase are unlikely to have changed the findings from the current study.

### Perspectives and significance

The present study found that sex and gender identity category, but not gender expression groups, influenced novel and traditional risk factors for CVD in a young, healthy cisgender population. Specifically, blood pressure (SBP, MAP) and central PWV were elevated in males compared to females, but %FMD, once larger artery diameter in males was controlled for, was improved in males compared to females. While young otherwise healthy males appear to have elevated measures of central stiffness, this may be compensated by elevated vasodilatory capacity of a major conduit artery (brachial artery). Given that this study is only generalizable to a young, healthy population of primarily university students of Asian and European/Caucasian racial and ethnic origins, further research is necessary to examine sex and gender considerations in other more ethnically diverse and representative groups of young adults, older adults, and those with CVD. Similarly, further research on populations of gender-diverse adults, including non-binary and transgender populations is warranted. Finally, further research is also needed examining participants in conditions where gender expression is challenged (i.e., masculinity stressors) or in populations that may experience gender-related stress (i.e., non-binary and transgender participants) and their influence on novel and traditional risk factors for CVD.

## Data Availability

The datasets used during the current study are available from the corresponding author on reasonable request. The Github package used for allometric scaling of flow–mediated dilation can be found here: https://github.com/jcherubini/Rtery.

## Supplementary material

Supplementary Figure S1 Relationships between %FMD or central PWV and BSRI-Masculine, BSRI-Feminine, and V̇O_2_peak. (**A**) Relationship between %FMD and BSRI-Masculine. (**B**) Relationship between central PWV and BSRI-Masculine. (**C**) Relationship between %FMD and BSRI-Feminine. (**D**) Relationship between central PWV and BSRI-Feminine. (**E**) Relationship between %FMD and V̇O_2_peak. (**F**) Relationship between central PWV and V̇O_2_peak; (**G**) Relationship between %FMD and baseline diameter.

Supplementary Table S1 Bem Sex Role Inventory Questionnaire.

Supplementary Table S2 Ethnicity and race of participants.

## References

[1] Vos T Lim SS Abbafati C Abbas KM Abbasi M Abbasifard M Global burden of 369 diseases and injuries in 204 countries and territories, 1990–2019: a systematic analysis for the Global Burden of Disease Study 2019. Lancet. (2020) 396:1204–22. 10.1016/S0140-6736(20)30925-9 33069326 PMC7567026

[2] Mensah GA Roth GA Fuster V. The global burden of cardiovascular diseases and risk factors. J Am Coll Cardiol. (2019) 74:2529–32. 10.1016/j.jacc.2019.10.009 31727292

[3] Aggarwal NR Patel HN Mehta LS Sanghani RM Lundberg GP Lewis SJ Sex differences in ischemic heart disease. Circ Cardiovasc Qual Outcomes. (2018) 11:e004437. 10.1161/CIRCOUTCOMES.117.004437 29449443

[4] Norris CM Yip CYY Nerenberg KA Clavel MA Pacheco C Foulds HJA State of the science in women’s cardiovascular disease: a Canadian perspective on the influence of sex and gender. J Am Heart Assoc. (2020) 9:e015634. 10.1161/JAHA.119.015634 32063119 PMC7070224

[5] Vogel B Acevedo M Appelman Y Bairey Merz CN Chieffo A Figtree GA The lancet women and cardiovascular disease commission: reducing the global burden by 2030. Lancet. (2021) 397:2385–438. 10.1016/S0140-6736(21)00684-X 34010613

[6] Pacheco C Mullen K-A Coutinho T Jaffer S Parry M Van Spall HGC The Canadian women’s heart health alliance atlas on the epidemiology, diagnosis, and management of cardiovascular disease in women—chapter 5: sex- and gender-unique manifestations of cardiovascular disease. CJC Open. (2022) 4:243–62. 10.1016/j.cjco.2021.11.006 35386135 PMC8978072

[7] Anand SS Islam S Rosengren A Franzosi MG Steyn K Yusufali AH Risk factors for myocardial infarction in women and men: insights from the INTERHEART study. Eur Heart J. (2008) 29:932–40. 10.1093/eurheartj/ehn018 18334475

[8] Moreau KL Stauffer BL Kohrt WM Seals DR. Essential role of estrogen for improvements in vascular endothelial function with endurance exercise in postmenopausal women. J Clin Endocrinol Metab. (2013) 98:4507–15. 10.1210/jc.2013-2183 24092827 PMC3816259

[9] Seals DR Jablonski KL Donato AJ. Aging and vascular endothelial function in humans. Clin Sci (Lond). (2011) 120:357–75. 10.1042/CS20100476 21244363 PMC3482987

[10] Wang C Lê-Scherban F Taylor J Salmoirago-Blotcher E Allison M Gefen D Associations of job strain, stressful life events, and social strain with coronary heart disease in the women’s health initiative observational study. J Am Heart Assoc. (2021) 10(5):e017780. 10.1161/JAHA.120.017780 33618543 PMC8174284

[11] Inaba Y Chen JA Bergmann SR. Prediction of future cardiovascular outcomes by flow-mediated vasodilatation of brachial artery: a meta-analysis. Int J Cardiovasc Imaging. (2010) 26:631–40. 10.1007/s10554-010-9616-1 20339920

[12] Holder SM Bruno RM Shkredova DA Dawson EA Jones H Hopkins ND Reference intervals for brachial artery flow-mediated dilation and the relation with cardiovascular risk factors. Hypertension. (2021) 77:1469–80. 10.1161/HYPERTENSIONAHA.120.15754 33745297

[13] Skaug E-A Aspenes ST Oldervoll L Mørkedal B Vatten L Wisløff U Age and gender differences of endothelial function in 4,739 healthy adults: the HUNT3 fitness study. Eur J Prev Cardiol. (2013) 20:531–40. 10.1177/2047487312444234 22456692

[14] Shenouda N Priest SE Rizzuto VI MacDonald MJ. Brachial artery endothelial function is stable across a menstrual and oral contraceptive pill cycle but lower in premenopausal women than in age-matched men. Am J Physiol Heart Circ Physiol. (2018) 315:H366–74. 10.1152/ajpheart.00102.2018 29727219

[15] Green DJ Hopkins ND Jones H Thijssen DH Eijsvogels TM Yeap BB. Sex differences in vascular endothelial function and health in humans: impacts of exercise. Exp Physiol. (2016) 101:230–42. 10.1113/EP085367 26663420

[16] Maruhashi T Soga J Fujimura N Idei N Mikami S Iwamoto Y Relationship between flow-mediated vasodilation and cardiovascular risk factors in a large community-based study. Heart. (2013) 99:1837–42. 10.1136/heartjnl-2013-304739 24153417 PMC3841746

[17] DuPont JJ Kenney RM Patel AR Jaffe IZ. Sex differences in mechanisms of arterial stiffness. Br J Pharmacol. (2019) 176:4208–25. 10.1111/bph.14624 30767200 PMC6877796

[18] London GM Guerin AP Pannier B Marchais SJ Stimpel M. Influence of sex on arterial hemodynamics and blood pressure. Hypertension. (1995) 26:514–9. 10.1161/01.HYP.26.3.514 7649591

[19] Ogola BO Zimmerman MA Clark GL Abshire CM Gentry KM Miller KS New insights into arterial stiffening: does sex matter? Am J Physiol Heart Circ Physiol. (2018) 315:H1073–87. 10.1152/ajpheart.00132.2018 30028199 PMC6415742

[20] Priest SE Shenouda N MacDonald MJ. Effect of sex, menstrual cycle phase, and monophasic oral contraceptive pill use on local and central arterial stiffness in young adults. Am J Physiol Heart Circ Physiol. (2018) 315:H357–65. 10.1152/ajpheart.00039.2018 29677465 PMC6139630

[21] Baldo MP Cunha RS Molina MdCB Chór D Griep RH Duncan BB Carotid-femoral pulse wave velocity in a healthy adult sample: the ELSA-Brasil study. Int J Cardiol. (2018) 251:90–5. 10.1016/j.ijcard.2017.10.075 29111104

[22] Collier SR Frechette V Sandberg K Schafer P Ji H Smulyan H Sex differences in resting hemodynamics and arterial stiffness following 4 weeks of resistance versus aerobic exercise training in individuals with pre-hypertension to stage 1 hypertension. Biol Sex Differ. (2011) 2:9. 10.1186/2042-6410-2-9 21867499 PMC3184039

[23] Lew LA Williams JS Stone JC Au AKW Pyke KE MacDonald MJ. Examination of sex-specific participant inclusion in exercise physiology endothelial function research: a systematic review. Front Sports Act Living. (2022) 4:860356. 10.3389/fspor.2022.860356 35399599 PMC8990239

[24] Tannenbaum C Ellis RP Eyssel F Zou J Schiebinger L. Sex and gender analysis improves science and engineering. Nature. (2019) 575:137–46. 10.1038/s41586-019-1657-6 31695204

[25] Tannenbaum C Greaves L Graham ID. Why sex and gender matter in implementation research. BMC Med Res Methodol. (2016) 16:145. 10.1186/s12874-016-0247-7 27788671 PMC5084413

[26] Krieger N. Genders, sexes, and health: what are the connections—and why does it matter? Int J Epidemiol. (2003) 32:652–7. 10.1093/ije/dyg156 12913047

[27] Mauvais-Jarvis F Bairey Merz N Barnes PJ Brinton RD Carrero J-J DeMeo DL Sex and gender: modifiers of health, disease, and medicine. Lancet. (2020) 396:565–82. 10.1016/S0140-6736(20)31561-0 32828189 PMC7440877

[28] Donnelly K Twenge JM. Masculine and feminine traits on the Bem Sex-Role Inventory, 1993–2012: a cross-temporal meta-analysis. Sex Roles. (2017) 76:556–65. 10.1007/s11199-016-0625-y

[29] Lee S Colditz GA Berkman LF Kawachi I. Caregiving and risk of coronary heart disease in U.S. women: a prospective study. Am J Prev Med. (2003) 24:113–9. 10.1016/S0749-3797(02)00582-2 12568816

[30] von Känel R Mausbach BT Patterson TL Dimsdale JE Aschbacher K Mills PJ Increased Framingham coronary heart disease risk score in dementia caregivers relative to non-caregiving controls. Gerontology. (2008) 54:131–7. 10.1159/000113649 18204247

[31] Parry M. Caregiver burden and cardiovascular disease: can we afford to keep the health of caregivers in Canada invisible? Can J Cardiol. (2019) 35:1267–9. 10.1016/j.cjca.2019.06.025 31515086

[32] Mortensen J Dich N Lange T Ramlau-Hansen CH Head J Kivimäki M Weekly hours of informal caregiving and paid work, and the risk of cardiovascular disease. Eur J Public Health. (2017) 28:743–7. 10.1093/eurpub/ckx227 PMC6051450 29309571

[33] Doster JA Purdum MB Martin LA Goven AJ Moorefield R. Gender differences, anger expression, and cardiovascular risk. J Nerv Ment Dis. (2009) 197(7):552–4. 10.1097/NMD.0b013e3181aac81b 19597365

[34] Pelletier R Khan NA Cox J Daskalopoulou SS Eisenberg MJ Bacon SL Sex versus gender-related characteristics: which predicts outcome after acute coronary syndrome in the young? J Am Coll Cardiol. (2016) 67:127–35. 10.1016/j.jacc.2015.10.067 26791057

[35] Williams JS Dunford EC Cheng JL Moncion K Valentino SE Droog CA The impact of the 24-h movement spectrum on vascular remodeling in older men and women: a review. Am J Physiol Heart Circ Physiol. (2021) 320:H1136–55. 10.1152/ajpheart.00754.2020 33449851

[36] Seeland U Nemcsik J Lønnebakken MT Kublickiene K Schluchter H Park C Sex and gender aspects in vascular ageing—focus on epidemiology, pathophysiology, and outcomes. Heart Lung Circ. (2021) 30:1637–46. 10.1016/j.hlc.2021.07.006 34452844

[37] Greaves L Ritz SA. Sex, gender and health: mapping the landscape of research and policy. Int J Environ Res Public Health. (2022) 19(5):2563. 10.3390/ijerph19052563 PMC8909483 35270255

[38] Lindsey ML Carter JR Ripplinger CM Kassiri Z Hansell Keehan K Brunt KR Sex still matters in cardiovascular research. Am J Physiol Heart Circ Physiol. (2022) 324:H79–81. 10.1152/ajpheart.00643.2022 36487186 PMC9799132

[39] Diaz-Canestro C Pentz B Sehgal A Montero D. Sex differences in cardiorespiratory fitness are explained by blood volume and oxygen carrying capacity. Cardiovasc Res. (2021) 118:334–43. 10.1093/cvr/cvab028 33538810

[40] Etsuda H Takase B Uehata A Kusano H Hamabe A Kuhara R Morning attenuation of endothelium-dependent, flow-mediated dilation in healthy young men: possible connection to morning peak of cardiac events? Clin Cardiol. (1999) 22:417–21. 10.1002/clc.4960220610 10376182 PMC6656222

[41] Canada S. Statistics Canada. Appendix 2.5: Ethnic or cultural origins disseminated in 2021, 2016, and 2011. In: Dictionary, Census of Population 2021. Ottawa ON, Canada: Government of Canada (2022). Available online at: https://www12.statcan.gc.ca/census-recensement/2021/ref/dict/app/index-eng.cfm?ID=a2_5 (Accessed November 24, 2022).

[42] Ontario Government. Government of Ontario. Collection of personal information: collection of personal information on race. In: Data Standards for the Identification and Monitoring of Systemic Racism. Toronto, ON, Canada: Government of Ontario (2022). p. 1–83. Available online at: https://www.ontario.ca/document/data-standards-identification-and-monitoring-systemic-racism/collection-personal-information#section-8 (Accessed November 24, 2022).

[43] Lagos D Compton D. Evaluating the use of a two-step gender identity measure in the 2018 general social survey. Demography. (2021) 58:763–72. 10.1215/00703370-8976151 33834217 PMC9084897

[44] Bauer GR Braimoh J Scheim AI Dharma C. Transgender-inclusive measures of sex/gender for population surveys: mixed-methods evaluation and recommendations. PLoS One. (2017) 12(5):e0178043. 10.1371/journal.pone.0178043 28542498 PMC5444783

[45] Bem SL. Bem Sex-Role Inventory: Professional Manual. Palo Alto, CA: Consulting Psychologists, Presse Medicale (1981).

[46] Bem SL. The measurement of psychological androgyny. J Consult Clin Psychol. (1974) 42:155–62. 10.1037/h0036215 4823550

[47] Yanico BJ. BSRI scores: stability over four years for college women. Psychol Women Q. (1985) 9:277–83. 10.1111/j.1471-6402.1985.tb00878.x

[48] Pelletier R Ditto B Pilote L. A composite measure of gender and its association with risk factors in patients with premature acute coronary syndrome. Psychosom Med. (2015) 77:517–26. 10.1097/PSY.0000000000000186 25984818

[49] Carver LF Vafaei A Guerra R Freire A Phillips SP. Gender differences: examination of the 12-item Bem Sex Role Inventory (BSRI-12) in an older Brazilian population. PLoS One. (2013) 8:e76356. 10.1371/journal.pone.0076356 24098482 PMC3788731

[50] Spence JT Helmreich R Stapp J. Ratings of self and peers on sex role attributes and their relation to self-esteem and conceptions of masculinity and femininity. J Pers Soc Psychol. (1975) 32:29–39. 10.1037/h0076857 1206468

[51] Van Bortel LM Laurent S Boutouyrie P Chowienczyk P Cruickshank JK De Backer T Expert consensus document on the measurement of aortic stiffness in daily practice using carotid-femoral pulse wave velocity. J Hypertens. (2012) 30:445–8. 10.1097/HJH.0b013e32834fa8b0 22278144

[52] Adkisson EJ Casey DP Beck DT Gurovich AN Martin JS Braith RW. Central, peripheral and resistance arterial reactivity: fluctuates during the phases of the menstrual cycle. Exp Biol Med (Maywood). (2010) 235:111–8. 10.1258/ebm.2009.009186 20404025 PMC2967291

[53] Williams JS Stimpson TV Tremblay JC Fenuta AM Pyke KE. No impact of acute hyperglycaemia on arterial stiffness in the early and late follicular phases of the menstrual cycle in young females. Exp Physiol. (2020) 105:174–83. 10.1113/EP087899 31628691

[54] Koivistoinen T Lyytikäinen L-P Aatola H Luukkaala T Juonala M Viikari J Pulse wave velocity predicts the progression of blood pressure and development of hypertension in young adults. Hypertension. (2018) 71:451–6. 10.1161/HYPERTENSIONAHA.117.10368 29311251

[55] Pyke KE Hartnett JA Tschakovsky ME. Are the dynamic response characteristics of brachial artery flow-mediated dilation sensitive to the magnitude of increase in shear stimulus? J Appl Physiol (1985). (2008) 105:282–92. 10.1152/japplphysiol.01190.2007 18467554

[56] Thijssen DHJ Bruno RM van Mil A Holder SM Faita F Greyling A Expert consensus and evidence-based recommendations for the assessment of flow-mediated dilation in humans. Eur Heart J. (2019) 40:2534–47. 10.1093/eurheartj/ehz350 31211361

[57] Wendelhag I Liang Q Gustavsson T Wikstrand J. A new automated computerized analyzing system simplifies readings and reduces the variability in ultrasound measurement of intima-media thickness. Stroke. (1997) 28:2195–200. 10.1161/01.STR.28.11.2195 9368564

[58] Shenouda N Gillen JB Gibala MJ MacDonald MJ. Changes in brachial artery endothelial function and resting diameter with moderate-intensity continuous but not sprint interval training in sedentary men. J Appl Physiol (1985). (2017) 123:773–80. 10.1152/japplphysiol.00058.2017 28546466 PMC5668454

[59] Myers J Arena R Franklin B Pina I Kraus WE McInnis K Recommendations for clinical exercise laboratories: a scientific statement from the American Heart Association. Circulation. (2009) 119:3144–61. 10.1161/CIRCULATIONAHA.109.192520 19487589

[60] Gillen JB Percival ME Skelly LE Martin BJ Tan RB Tarnopolsky MA Three minutes of all-out intermittent exercise per week increases skeletal muscle oxidative capacity and improves cardiometabolic health. PLoS One. (2014) 9:e111489. 10.1371/journal.pone.0111489 25365337 PMC4218754

[61] Cohen J. Statistical Power Analysis for the Behavioral Sciences. New York, NY: Routledge (1988).

[62] Rosenthal R. Parametric Measures of Effect Size. The Handbook of Research Synthesis. New York, NY: Russell Sage Foundation (1994). p. 231–44.

[63] Atkinson G Batterham AM. The percentage flow-mediated dilation index: a large-sample investigation of its appropriateness, potential for bias and causal nexus in vascular medicine. Vasc Med. (2013) 18:354–65. 10.1177/1358863X13508446 24172228

[64] Atkinson G Batterham AM. Allometric scaling of diameter change in the original flow-mediated dilation protocol. Atherosclerosis. (2013) 226:425–7. 10.1016/j.atherosclerosis.2012.11.027 23261170

[65] Cherubini JM. Rtery: An R Package for Endothelial Function Analysis. GitHub (2023). Available online at: https://github.com/jcherubini/Rtery (Accessed Feburary 21, 2023).

[66] Cronbach LJ. Coefficient alpha and the internal structure of tests. Psychometrika. (1951) 16:297–334. 10.1007/BF02310555

[67] Tavakol M Dennick R. Making sense of Cronbach’s alpha. Int J Med Educ. (2011) 2:53–5. 10.5116/ijme.4dfb.8dfd 28029643 PMC4205511

[68] Streiner DL. Starting at the beginning: an introduction to coefficient alpha and internal consistency. J Pers Assess. (2003) 80:99–103. 10.1207/S15327752JPA8001_18 12584072

[69] Maney DL. Perils and pitfalls of reporting sex differences. Philos Trans R Soc Lond B Biol Sci. (2016) 371:20150119. 10.1098/rstb.2015.0119 26833839 PMC4785904

[70] Harris RA Tedjasaputra V Zhao J Richardson RS. Premenopausal women exhibit an inherent protection of endothelial function following a high-fat meal. Reprod Sci. (2012) 19:221–8. 10.1177/1933719111418125 22383760 PMC3343134

[71] Marlatt KL Kelly AS Steinberger J Dengel DR. The influence of gender on carotid artery compliance and distensibility in children and adults. J Clin Ultrasound. (2013) 41:340–6. 10.1002/jcu.22015 23233368 PMC3736987

[72] Vermeersch SJ Rietzschel ER De Buyzere ML De Bacquer D De Backer G Van Bortel LM Age and gender related patterns in carotid-femoral PWV and carotid and femoral stiffness in a large healthy, middle-aged population. J Hypertens. (2008) 26:1411–9. 10.1097/HJH.0b013e3282ffac00 18551018

[73] Sun P Chen X Zeng Z Li S Wang J Yu F Sex differences in lower-limb arterial stiffness following acute aerobic exercise. Sci Sports. (2020) 35:e39–48. 10.1016/j.scispo.2019.02.007

[74] Holder SM Brislane A Dawson EA Hopkins ND Hopman MTE Cable NT Relationship between endothelial function and the eliciting shear stress stimulus in women: changes across the lifespan differ to men. J Am Heart Assoc. (2019) 8(4):e010994. 10.1161/JAHA.118.010994 30764688 PMC6405684

[75] Ali Hamza M Abdulhameed A Ali Mansour A. Total testosterone to estradiol ratio as a predictor marker of metabolic syndrome in males. Arch Razi Inst. (2022) 77(1):351–7. 10.22092/ari.2021.356607.1878 35891738 PMC9288628

[76] Khaw KT Barrett-Connor E. Blood pressure and endogenous testosterone in men: an inverse relationship. J Hypertens. (1988) 6(4):329–32. 3379300

[77] Dubey RK Oparil S Imthurn B Jackson EK. Sex hormones and hypertension. Cardiovasc Res. (2002) 53:688–708. 10.1016/S0008-6363(01)00527-2 11861040

[78] Reckelhoff JF. Gender differences in the regulation of blood pressure. Hypertension. (2001) 37:1199–208. 10.1161/01.HYP.37.5.1199 11358929

[79] Choi JY Yun EK Yeun EJ Jeong ES. Factors influencing blood pressure classification for adults: gender differences. Int J Nurs Pract. (2019) 25:e12706. 10.1111/ijn.12706 30450629

[80] Black MA Cable NT Thijssen DHJ Green DJ. Impact of age, sex, and exercise on brachial artery flow-mediated dilatation. Am J Physiol Heart Circ Physiol. (2009) 297:H1109–16. 10.1152/ajpheart.00226.2009 19633208 PMC2755978

[81] Vlachopoulos C Aznaouridis K Stefanadis C. Prediction of cardiovascular events and all-cause mortality with arterial stiffness: a systematic review and meta-analysis. J Am Coll Cardiol. (2010) 55:1318–27. 10.1016/j.jacc.2009.10.061 20338492

[82] Levenson J Pessana F Gariepy J Armentano R Simon A. Gender differences in wall shear-mediated brachial artery vasoconstriction and vasodilation. J Am Coll Cardiol. (2001) 38:1668–74. 10.1016/S0735-1097(01)01604-7 11704379

[83] Mizia-Stec K Gasior Z Mizia M Haberka M Holecki M Zwolińska W Flow-mediated dilation and gender in patients with coronary artery disease: arterial size influences gender differences in flow-mediated dilation. Echocardiography. (2007) 24:1051–7. 10.1111/j.1540-8175.2007.00531.x 18001358

[84] Tremblay JC Stimpson TV Pyke KE. Evidence of sex differences in the acute impact of oscillatory shear stress on endothelial function. J Appl Physiol. (2018) 126:314–21. 10.1152/japplphysiol.00729.2018 30382805 PMC6397415

[85] Celermajer DS Sorensen KE Spiegelhalter DJ Georgakopoulos D Robinson J Deanfield JE. Aging is associated with endothelial dysfunction in healthy men years before the age-related decline in women. J Am Coll Cardiol. (1994) 24:471–6. 10.1016/0735-1097(94)90305-0 8034885

[86] Mollica MA Smith AW Kent EE. Caregiving tasks and unmet supportive care needs of family caregivers: a U.S. population-based study. Patient Educ Couns. (2020) 103:626–34. 10.1016/j.pec.2019.10.015 31704030

[87] Nielsen MW Stefanick ML Peragine D Neilands TB Ioannidis JPA Pilote L Gender-related variables for health research. Biol Sex Differ. (2021) 12:23. 10.1186/s13293-021-00366-3 33618769 PMC7898259

[88] Age, Sex at Birth and Gender Reference Guide, Census of Population. Ottawa ON, Canada: Statistics Canada. Government of Canada (2022).

[89] Poteat TC Divsalar S Streed CG Feldman JL Bockting WO Meyer IH. Cardiovascular disease in a population-based sample of transgender and cisgender adults. Am J Prev Med. (2021) 61:804–11. 10.1016/j.amepre.2021.05.019 34364725 PMC8608688

[90] Morgan E D’Aquila R Carnethon MR Mustanski B. Cardiovascular disease risk factors are elevated among a cohort of young sexual and gender minorities in Chicago. J Behav Med. (2019) 42:1073–81. 10.1007/s10865-019-00038-z 30968321 PMC6785361

[91] Mereish EH Goldstein CM. Minority stress and cardiovascular disease risk among sexual minorities: mediating effects of sense of mastery. Int J Behav Med. (2020) 27:726–36. 10.1007/s12529-020-09919-z 32734472 PMC7676398

[92] Longpré-Poirier C Dougoud J Jacmin-Park S Moussaoui F Vilme J Desjardins G Sex and gender and allostatic mechanisms of cardiovascular risk and disease. Can J Cardiol. (2022) 38:1812–27. 10.1016/j.cjca.2022.09.011 36150584

[93] Kramer BL Himmelstein MS Springer KW. Getting to the heart of masculinity stressors: masculinity threats induce pronounced vagal withdrawal during a speaking task. Ann Behav Med. (2017) 51:846–55. 10.1007/s12160-017-9907-z 28401414

[94] Burke PJ Cast AD. Stability and change in the gender identities of newly married couples. Soc Psychol Q. (1997) 60:277–90. 10.2307/2787090

[95] Mehta CM. Gender in context: considering variability in wood and Eagly’s traditions of gender identity. Sex Roles. (2015) 73:490–6. 10.1007/s11199-015-0535-4

[96] Fan P-L Marini MM. Influences on gender-role attitudes during the transition to adulthood. Soc Sci Res. (2000) 29:258–83. 10.1006/ssre.1999.0669

[97] Auster CJ Ohm SC. Masculinity and femininity in contemporary American society: a reevaluation using the Bem Sex-Role Inventory. Sex Roles. (2000) 43:499–528. 10.1023/A:1007119516728

[98] Hansson RO Chernovetz ME Jones WH. Maternal employment and androgyny. Psychol Women Q. (1977) 2:76–8. 10.1111/j.1471-6402.1977.tb00575.x

[99] Kerr P Da Torre MB Giguère C Lupien SJ Juster RP. Occupational gender roles in relation to workplace stress, allostatic load, and mental health of psychiatric hospital workers. J Psychosom Res. (2021) 142:110352. 10.1016/j.jpsychores.2020.110352 33450429

[100] Williams JS MacDonald MJ. Influence of hormonal contraceptives on peripheral vascular function and structure in premenopausal females: a review. Am J Physiol Heart Circ Physiol. (2021) 320:H77–89. 10.1152/ajpheart.00614.2020 33164574

[101] Williams MR Westerman RA Kingwell BA Paige J Blombery PA Sudhir K Variations in endothelial function and arterial compliance during the menstrual cycle. J Clin Endocrinol Metab. (2001) 86:5389–95. 10.1210/jcem.86.11.8013 11701712

[102] D'Urzo KA King TJ Williams JS Silvester MD Pyke KE. The impact of menstrual phase on brachial artery flow-mediated dilatation during handgrip exercise in healthy premenopausal women. Exp Physiol. (2018) 103:291–302. 10.1113/EP086311 29083061

[103] Hashimoto M Akishita M Eto M Ishikawa M Kozaki K Toba K Modulation of endothelium-dependent flow-mediated dilatation of the brachial artery by sex and menstrual cycle. Circulation. (1995) 92:3431–5. 10.1161/01.CIR.92.12.3431 8521564

[104] Williams JS Dunford EC MacDonald MJ. Impact of the menstrual cycle on peripheral vascular function in premenopausal women: systematic review & meta-analysis. Am J Physiol Heart Circ Physiol. (2020) 319:H1327–37. 10.1152/ajpheart.00341.2020 33064553

