# Supplementary material for: Differences in cardiovascular risk factors associated with sex and gender identity, but not gender expression, in young, healthy cisgender adults
Source: Front Cardiovasc Med. 2024 Sep 5;11:1374765. doi: 10.3389/fcvm.2024.1374765 (PMC11420989; doi:10.3389/fcvm.2024.1374765)
Supplement: Supplementary Table S2 — Ethnicity and race of participants. [file Datasheet1.pdf]

Supplementary Table 1 – Bem Sex Role Inventory Questionnaire

The following table includes the Bem Sex Role Inventory – 30 Questionnaire.

"Rate yourself on each item below, on a scale of 1 (Never or almost never true) to 7 (Almost always true)"

	1 – Never/Almost Never True	2	3	4	5	6	7 - Almost Always True
Defend my own beliefs							
Have leadership abilities							
Affectionate							
Eager to soothe hurt feelings							
Conscientious							
Secretive							
Independent							
Willing to take risks							
Sympathetic							
Warm							
Moody							
Adaptable							
Assertive							
Dominant							
Sensitive to the needs of others							
	1 – Never/Almost Never True	2	3	4	5	6	7 - Almost Always True
Tender							
Reliable							
Conceited							
Strong personality							
Willing to take a stand							
Understanding							
Loves children							
Jealous							
Tactful							
Forceful							
Aggressive							
Compassionate							
Gentle							
Truthful							
Conventional							

Supplementary Table 2 – Ethnicity & Race of Participants

Data is reported in alphabetical order:

Ethnicity of Participants	Race of Participants
African Origins (n=5, 4%)	Asian (n=48, 37%)
Asian Origins (n=54, 42%)	Black or African American (n=2, 2%)
European Origins (n=38, 29%)	Middle Eastern or North African (n=10, 8%)
North American Origins (n=3, 2%)	White or Caucasian (n=60, 46%)
Mixed Ethnicity (n=30, 23%)<br>n=1 African & Asian Origins<br>n=1 African & European Origins<br>n=2 Asian, Caribbean, & European Origins<br>n=7 Asian & European Origins<br>n=1 Asian, European & Latin American Origins<br>n=1 Asian, European & Other North American Origins<br>n=2 Asian & North American Origins<br>n=1 Canadian-Punjabi/South Asian Origins<br>n=1 Caribbean & European Origins<br>n=13 European & Other North American Origins	Mixed Race (n=8, 6%)<br>n=6 Asian & European/Caucasian<br>n=1 Aboriginal & Caucasian<br>n=1 Not disclosed<br><br>Prefer to Self-Disclose (n=2)<br>n=1 Brown<br>n=1 Brown/South Indian

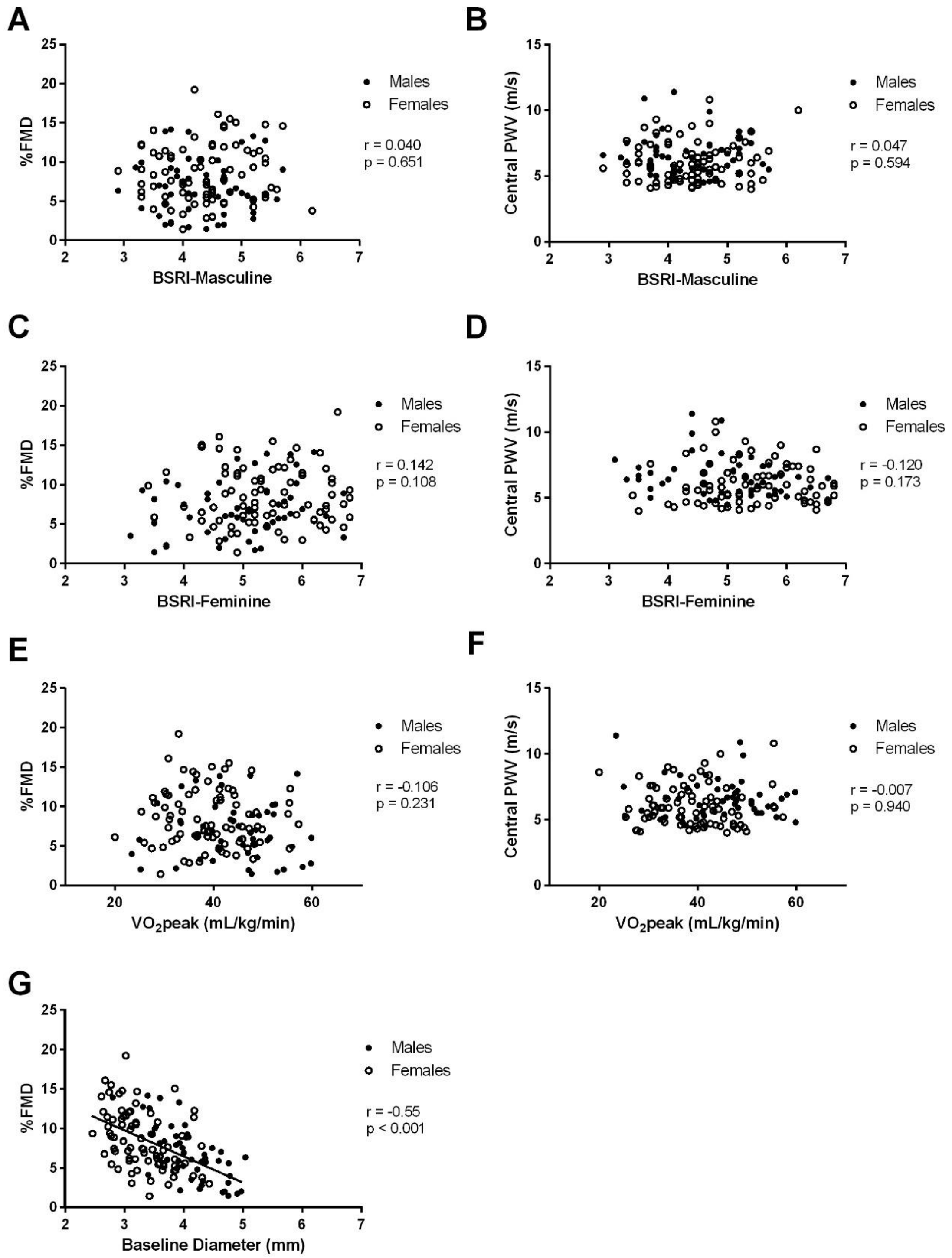

Supplementary Figure 1. Relationships between %FMD or Central PWV and BSRI-Masculine, BSRI-Feminine, and $\dot{V}O_2$peak. (A) Relationship between %FMD and BSRI-Masculine; (B) Relationship between Central PWV and BSRI-Masculine; (C) Relationship between %FMD and BSRI-Feminine; (D) Relationship between Central PWV and BSRI-Feminine; (E) Relationship between %FMD and $\dot{V}O_2$peak; (F) Relationship between Central PWV and $\dot{V}O_2$peak; (G) Relationship between %FMD and baseline diameter.
